# A double scrambling-DNA row and column closed loop image encryption algorithm based on chaotic system

**DOI:** 10.1371/journal.pone.0267094

**Published:** 2022-07-12

**Authors:** Weiyu Ran, Erfu Wang, Zhiyong Tong

**Affiliations:** 1 Key Lab of Electronic and Communication Engineering, Heilongjiang University, Harbin, China; 2 Electrical Engineering College, Heilongjiang University, Harbin, China; 3 Section of Data and Information, Heilongjiang Provincial Military Command, Harbin, China; Universiti Sains Malaysia, MALAYSIA

## Abstract

In this paper, a dynamic update algorithm of double scrambling-DNA row and column closed loop based on chaotic system is proposed. The classical scrambling and diffusion structure are used in the whole process. In the scrambling stage, a new pixel reconstruction method is proposed by combining the Hilbert curve with Knuth-Durstenfeld shuffle algorithm to overcome the shortcoming of nearby storage of Hilbert curve. This method reconstructs the pixel matrix of one-dimensional vector according to the Hilbert curve coding method, and achieves good scrambling effect, while reducing its time complexity and space complexity. In the diffusion stage, combining the plaintext row, the ciphertext row and the key row, and taking advantage of the parallel computing power and high storage density of the DNA encoding, the existing block diffusion operation is improved, and the two-round diffusion of the DNA encoding is proposed. When the last line of ciphertext is generated, the first line of ciphertext is updated and the closed-loop dynamic update of the encryption system is realized. Finally, SHA-256 is used to give the secret key and calculate the initial value of the chaotic system. The simulation results show that the “double scrambling-DNA row and column closed loop dynamic” update algorithm proposed in this paper can effectively improve the efficiency of information transmission and have high security.

## 1. Introduction

With the rapid development of network and science technology, image is widely used in social media, network, military, medical and other fields. However, while facing a large amount of information sharing, it is gradually facing hidden dangers such as data leakage, tampering and counterfeiting. In recent years, digital image encryption and its related technologies have been widely concerned by scholars. At present, a large number of research methods have been proposed, which apply compressed sensing theory [1, 2], optical theory [3, 4], chaos theory [5–7], cellular automata [8, 9], DNA operation coding [10, 11], and other techniques to image encryption.

The chaotic system has many characteristics that make it more suitable for image encryption, such as extreme sensitivity to initial values and reproducibility, and the chaotic system generates sequences very fast, so it has become a focus of attention in the direction of image encryption. It is undeniable that although chaos-based image encryption technology is more suitable than traditional encryption, there are still security risks. In [12], a cross-coupling multi-grayscale image encryption scheme based on two piecewise linear chaotic maps is proposed. Although the discreteness of chaotic system is improved, and the disadvantage of chaos diffusion is avoided by a single chaotic map, the range of control parameters of the system is often limited. Because the parameter is usually set as the key, there will be a very small key space, which is unsafe to the encryption algorithm. In [13], Hu and Li.proposed a coupled chaotic system based on a certain unit transformation. A two-way multi-round transformation network is designed, and the input image is divided into high bit and low bit for encryption. Although any two one-dimensional chaos are combined to generate a new one-dimensional chaos with better performance, it is low dimensional chaos is easy to implement and cannot meet the current demand for randomness. In [14], Kaur et al., in order to solve the hyperparameter problem of chaotic systems, proposed a chaotic search image encryption technology combining nondominated sorting genetic algorithm and local sorting algorithm. Because the programming of high-dimensional chaotic maps is difficult and takes a lot of time, although it can provide better random streams and data streams, it consumes too much resources and is not suitable for encryption schemes for ordinary images. Also, the initial value and parameter of the chaotic system are independent of the plaintext image, and it is less sensitive to the plaintext image. Aiming at the problems of poor randomness, difficulty in programming, and limited parameter range resulting in small key space in the chaotic dynamic system, this paper adopts a two-dimensional cascade modulation chaotic system (2D-LICM). This scheme links the pixel value of the plaintext image with the initial value of the chaotic system, and because the chaotic system is extremely sensitive to the initial value, the key space is increased. The chaotic spread spectrum sequence generated by the two-dimensional cascade coupled modulation chaotic system has excellent performance and high confidentiality, which is very suitable for image encryption.

Due to low-power and complementary rules of DNA base and because DNA has vast parallelism and extraordinary storage density, DNA computing also applies to image encryption. In [15], Zhu et al. discussed image encryption based on Kronecker product and DNA operations, mapping the pixel values of ordinary images to a finite field, and then using the Kronecker product matrix to scramble. Finally, further scrambling and diffusion are achieved using DNA manipulation. In [16], Chai et al. combined the DNA sequence with the hyperchaotic system and cellular automata, used the block diffusion method for the image, and combined the previous diffusion block image with the two-dimensional cellular automata to affect the encryption of the current block image. Although image encryption algorithm based on DNA has become a hot topic in recent years, there are also many encryption schemes that are not secure. In [17], a cryptanalysis of image encryption algorithm based on DNA and chaotic mapping is proposed. Firstly, the image pixels are transformed by DNA sequence, then added with the DNA matrix generated by the one-dimensional chaotic system. Finally, the image is divided into blocks, and the two chaotic sequences generated by the two-dimensional chaos are used for scrambling. The security of this algorithm depends on the initial conditions of one-dimensional and two-dimensional chaotic systems, but its initial conditions are composed of the first half pixel sum and the second half pixel sum of the original image. Since the attacker can know these parameters, this scheme is not secure enough. Aiming at this weakness, we use hash256 function of plaintext image to calculate the initial value of chaotic system. The proposed algorithm is highly sensitive to plaintext image and can resist the selected plaintext attack. In [18], a cracking scheme of image encryption algorithm based on DNA encoding and spatiotemporal chaos is proposed. The scheme uses the DNA-based arrangement to scramble the pixels of the image, but the DNA-based arrangement only changes the position, its diffusion part does not involve any key parameters, and the computational complexity of the single-round encryption algorithm is also very low. Aiming at this problem, we use the shuffling algorithm and Hilbert curve to achieve double dynamic scrambling, which greatly reduces the time and space complexity of the algorithm. In the diffusion part, we use the DNA mask generated by the chaotic system to diffuse the pixels, and the key is related to the plaintext image, which greatly improves the sensitivity of the plaintext image. In [19], cryptanalysis of chaotic image encryption scheme combining DNA and entropy was proposed. There are two vulnerabilities in this algorithm, firstly the entropy quoted cannot protect the scrambling operation under chosen-plaintext attack, because the entropy can be reconstructed directly from the password image; secondly, the replacement of the last column of pixels leaks the encoding rule pattern. In view of these two vulnerabilities, we propose some improvement schemes. Compared with using entropy, hash function is used to generate keys, which is very sensitive to the initial value. In the diffusion operation, each ciphertext consists of the current plaintext line, the key line and the ciphertext generated by the previous line, which form a complete diffusion system. In order to solve the security risks and low sensitivity to pure images of the above DNA algorithms, this paper proposes a closed-loop dynamic update algorithm for DNA encoding, which encodes the chaotic sequence and the scrambled cryptographic image separately. Use the closed-loop XOR method of plaintext lines, ciphertext lines, and key lines, and update the first line of ciphertext after the last line of ciphertext is generated, to achieve higher security of the encryption system. The random sequence generated by the chaotic system and the scrambled image XOR have better randomness and complexity. The use of the parallel computing power and huge storage capacity of DNA coding improves the encryption speed of this article.

Based on the above discussion, this paper proposes a new image encryption technology based on a two-dimensional cascade modulation chaotic system (2D-LICM). The proposed image scrambling technology adopts double scrambling: combining the Hilbert curve and Knuth-Durstenfeld shuffle algorithm to implement the closed-loop update scrambling algorithm of image pixels from n-dimension to 1-dimension, and then from 1-dimension to n-dimension. The effective diffusion technology uses DNA coding to perform row and column closed-loop dynamic diffusion. Each ciphertext is constructed by the current plaintext line, the key line and the ciphertext generated by the previous line. When the last line of ciphertext is generated, the first line of ciphertext is updated, which realizes the closed-loop dynamic diffusion technology. DNA coding, plaintext, ciphertext and key stream form a complete system, so as to ensure the security of the encryption system. In addition, using the hash 256 function of the plaintext image to generate the parameter of the two-dimensional cascade modulation chaotic system, it can be seen that the algorithm proposed in this paper is highly sensitive to the plaintext image and can resist the selected plaintext attack.

The contributions of this paper are as follows:

1. The double scrambling method is proposed to increase the randomness between image pixels and the security of encryption scheme.
2. We propose a DNA-row-column closed-loop dynamic update diffusion scheme, which combines the plaintext, ciphertext and key into a complete system.
3. The initial value of the chaotic system is closely related to the plaintext, so that the encryption algorithm has a large enough key space to resist attacks.
4. Dynamic DNA coding has huge parallelism, which improves encryption time and efficiency.

The rest of this paper is organized as follows: Section 2 presents the relevant theoretical basis. Section 3 describes the double scrambling-DNA row and column closed loop dynamic encryption algorithm. Section 4 introduces the simulation experiment and security analysis of the algorithm. Section 5 presents the discussion and prospects. Finally, Section 6 summarizes the paper.

## 2. Relevant theoretical basis

### 2.1 2D-LICM chaotic system

The logistic chaotic system and ICMIC are common one-dimensional chaotic maps. Two-dimensional cascade modulation chaotic system (2D-LICM) [20] are obtained through the cascade modulation coupling model. The generated sequence is more random and suitable for image encryption. Two-dimensional cascade modulation chaotic system (2D-LICM) is defined by the following Eq (1):

$$\begin{array}{c} \left\{ \begin{array}{cc} & x_{i+1}=\text{sin}(21/(a(y_{i}+3)kx_{i}(1-kx_{i})))\\ & y_{i+1}=\text{sin}(21/(a(kx_{i+1}+3)y_{i}(1-y_{i}))) \end{array}\right. \end{array}$$ (1)

**Fig 1:**
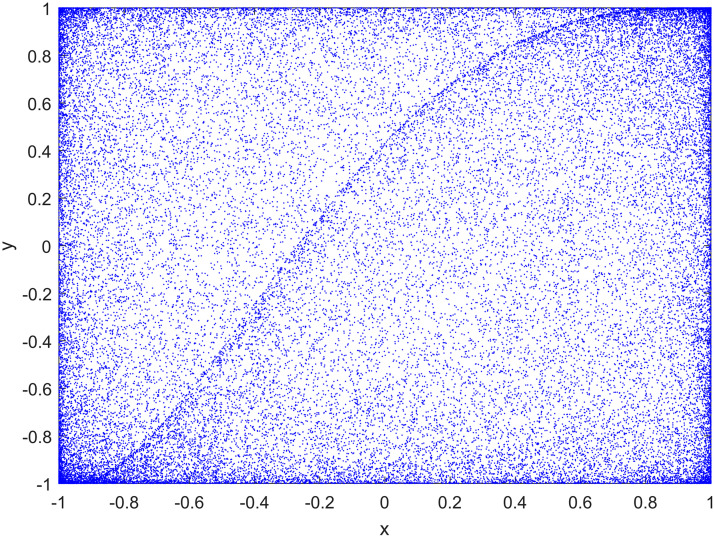
Attractors of 2D-LICM chaotic system with (*a*, *k*) = (0.6, 0.8).

**Fig 2:**
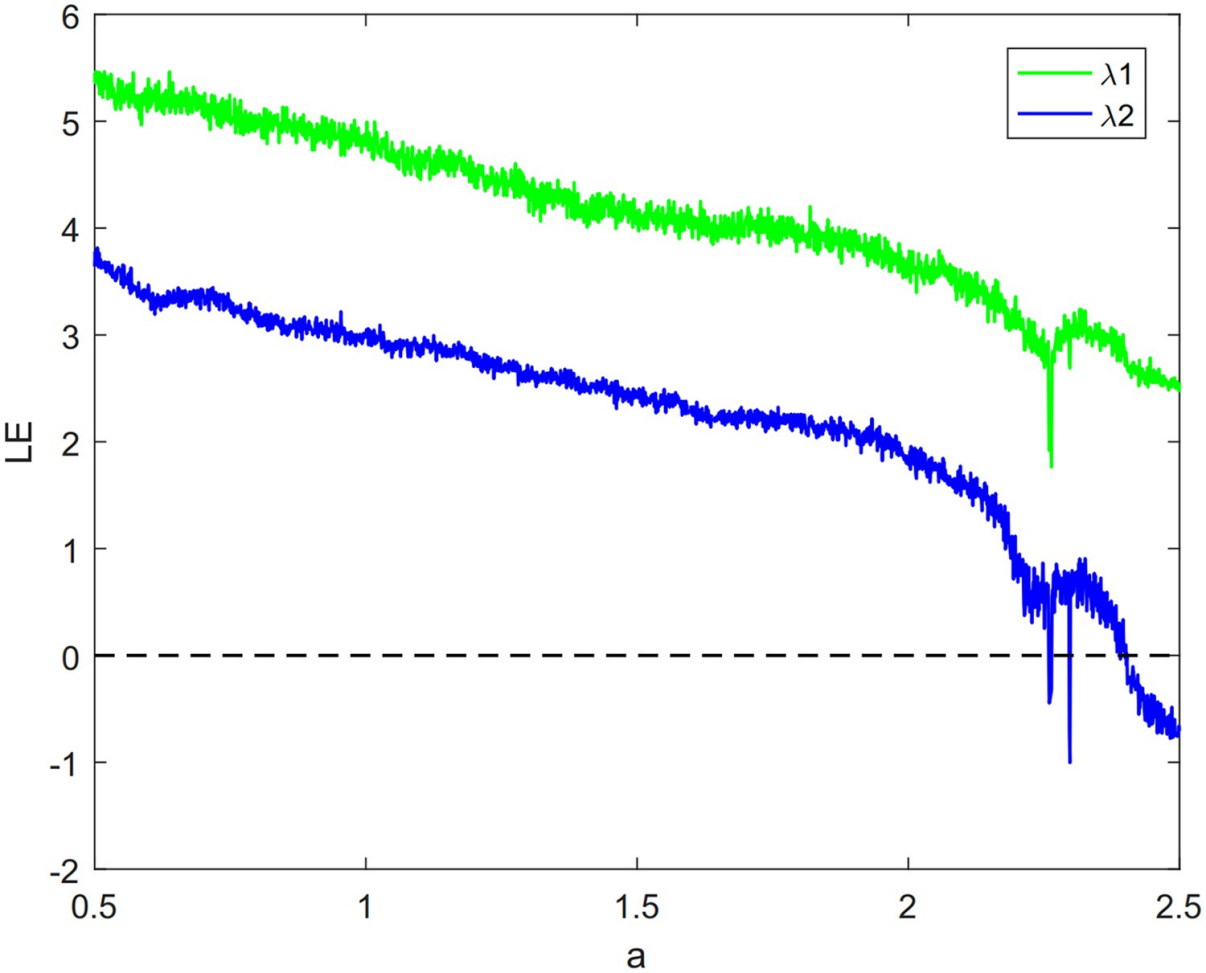
Lyapunov spectrum of 2D-LICM chaotic system with *a* from 0.5 to 2.5.

**Fig 3:**
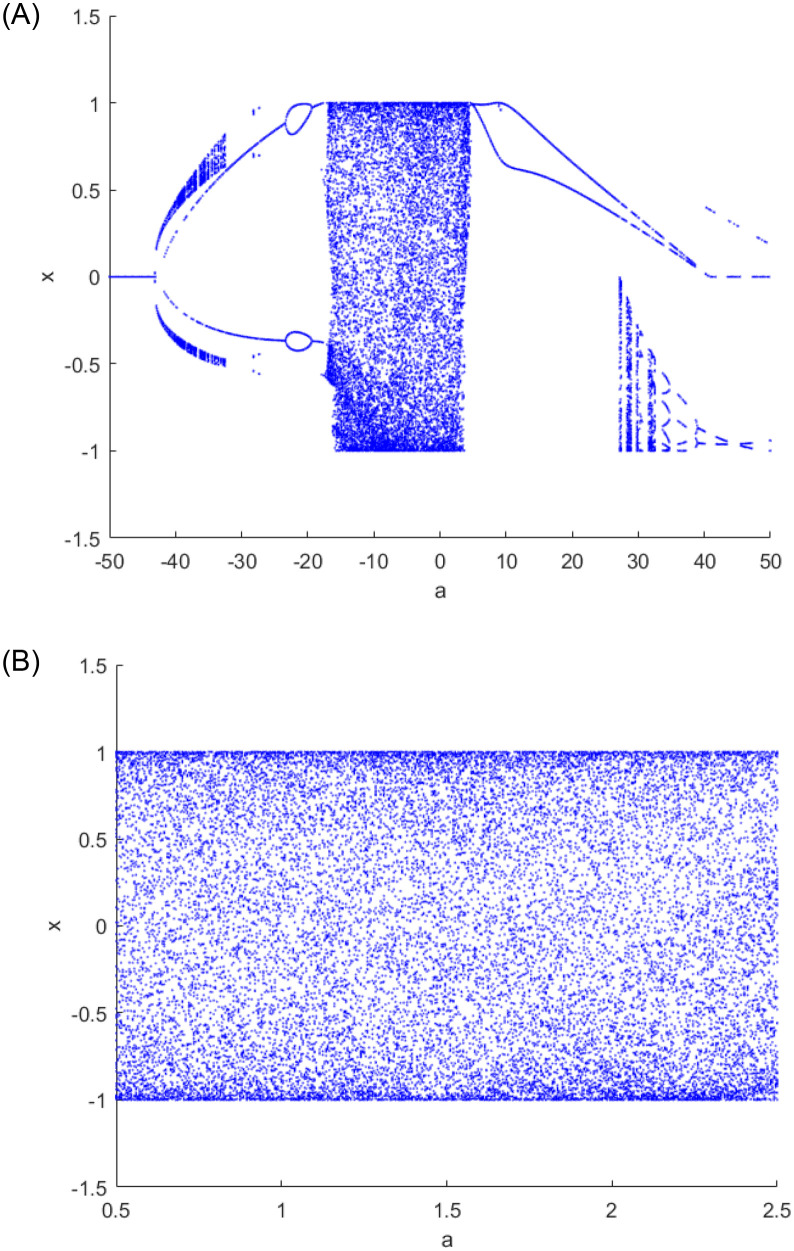
Bifurcation diagram of the 2D-LICM chaotic system. A: Bifurcation diagram of *a* ∈ [−50, 50] (*k* = 0.8). B: Bifurcation diagram of *a* ∈ [0.5, 2.5] (*k* = 0.8).

Among them, *a* and *k* are system parameters, *a* ∈ (0, ∞), *k* ∈ (0, ∞). Attractors of two-dimensional cascade modulation chaotic system (2D-LICM) with (*a*, *k*) = (0.6, 0.8) is shown in Fig 1. It can be seen from the Fig 1 that 2D-LICM has better ergodicity and randomness. Lyapunov exponents (LEs) are an important index to evaluate the chaotic characteristics of the system. In Fig 2, when *k* = 0.8 and *a* varies from 0.5 to 2.5, the system is hyperchaotic. The bifurcation diagram describes the state of the system when it changes with a certain system parameter or initial value. By observing the bifurcation diagram, we can easily judge whether the system is in chaos state or other state, and reveal the influence of a certain parameter or initial value on the system state. As can be seen from Fig 3(A), when *k* = 0.8 and the parameter range of *a* is [−50, 50], the system equilibrium point has obvious bifurcation change with the change of parameter *a*. And when the variation range of parameter *a* is 0.5 < *a* < 2.5, it can be clearly seen from Fig 3(B) that with the change of parameter *a*, the distribution of the system is relatively dense, the degree of chaos of the system remains within a certain range, and the change is not obvious, that is, the degree of chaos is in a relatively stable state.

### 2.2 Hilbert curve

**Fig 4:**
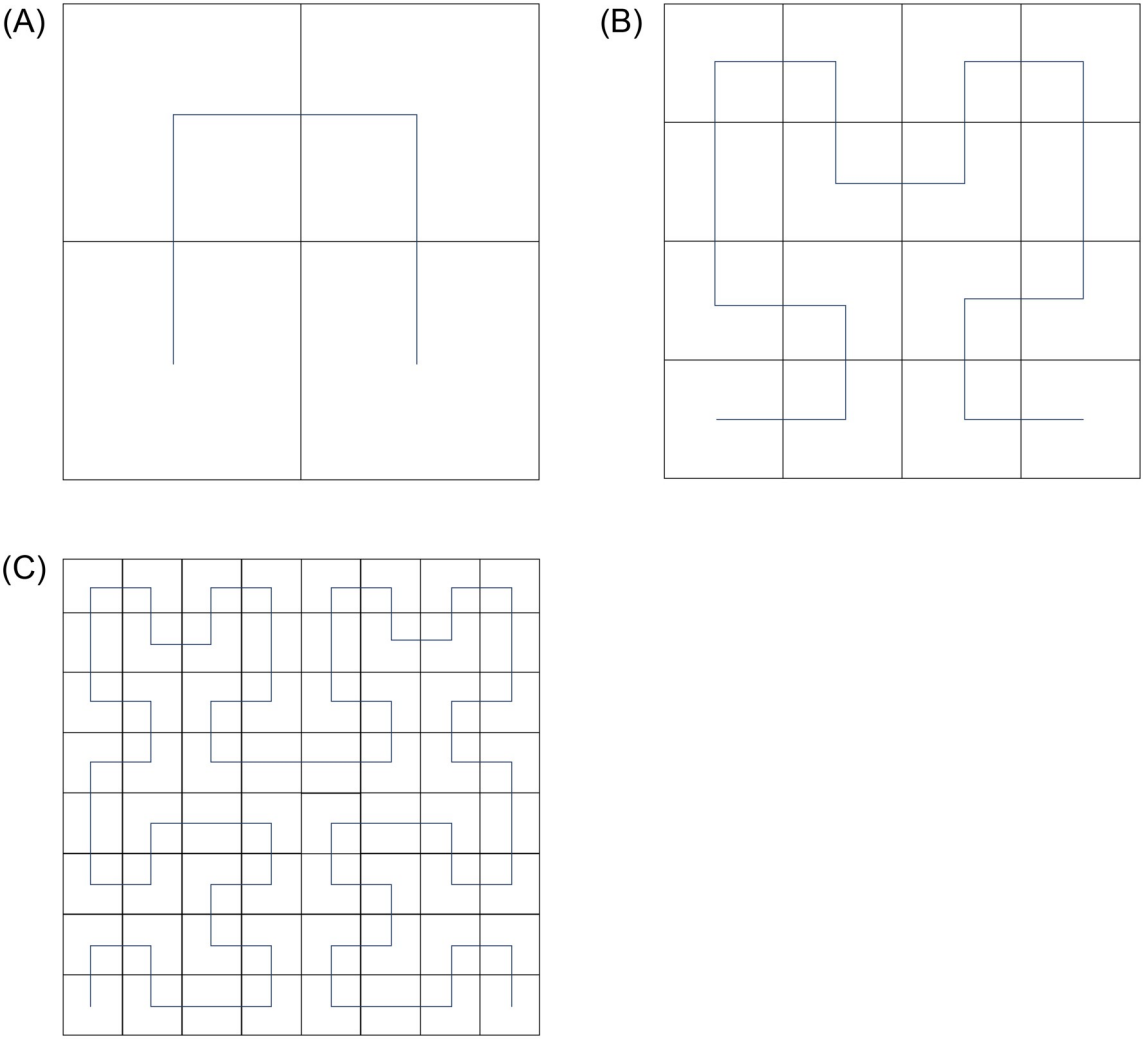
Hilbert curve of order 1, 2 and 3. A: Hilbert curve of order 1. B: Hilbert curve of order 2. C: Hilbert curve of order 3.

**Fig 5:**
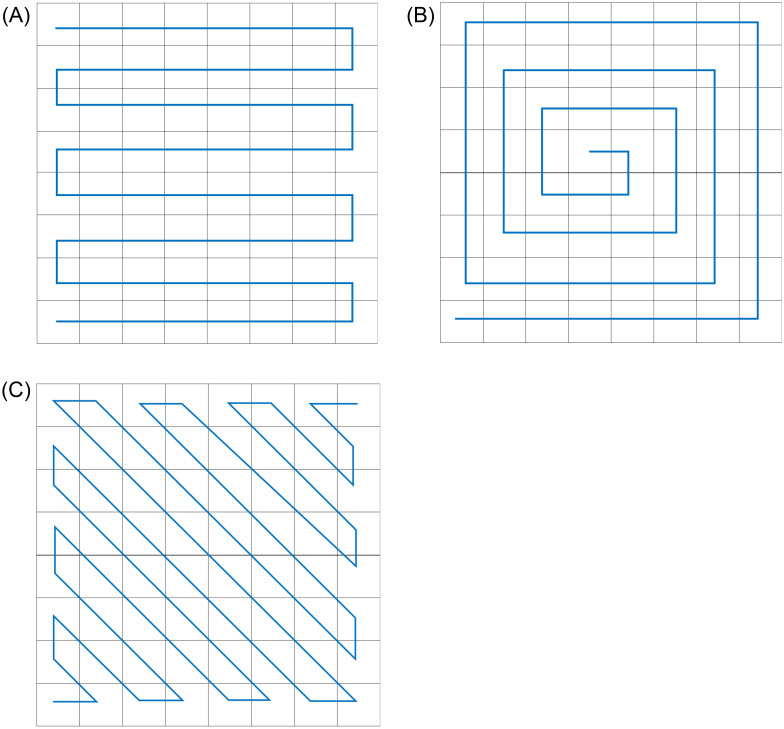
Different curves. A: Continuous raster method. B: Spiral method. C: Diagonal method.

Hilbert curve is a pattern of scanning 2^*n*^ × 2^*n*^ dot matrix [21], which is often used to scramble the pixel positions of the original image. According to the characteristics of its own space filling curve, it can linearly pass through each discrete unit of two-dimensional or higher dimensions, and only pass once. The curve can linearly sort and encode each discrete unit, which is the unique identification of the unit. The idea of curve construction is that any dimension can be abstracted as the splicing of four matrices, as shown in Fig 4, which are Hilbert curves of order 1, order 2 and order 3 respectively. According to different arrangement rules can obtain different scanning patterns, such as z-order curve, diagonal curve, and spiral curve [22], as shown in Fig 5.

### 2.3 Knuth-Durstenfeld shuffle algorithm

The Knuth-Durstenfeld shuffle algorithm [23] is based on generating finite random sequences and is a relatively effective shuffling algorithm at present. The algorithm improves on the classic shuffle algorithm of Fisher Yates [24] and scrambles the sorting sequence. It interacts with the numbers on the original array to save extra space. The basic idea of this algorithm is to randomly take a number from the unprocessed data each time and put it at the end of the array, that is, the tail of the array stores the processed numbers.

In this paper, the steps of the algorithm are as follows:

The sequence to be sorted is *C*[*n*], and function *rand*(*i*) outputs a uniform random integer between 1 and *i*.

Step 1: *i* = 1.
Step 2: *r* = *floor*(*rand* * *i*) + 1
Step 3: exchange *C*[*i*] and *C*[*r*].
Step 4: iteration Step 2 and Step 3, to *i* = *n*.

### 2.4 DNA encoding and XOR operation

DNA encoding is usually used in image encryption to utilize DNA as a carrier of the content to be encrypted, and makes base pair complementary substitution for a certain number of iterations to get the encrypted image. DNA coding is a double-stranded polymer compound, which consists of four deoxyribonucleic acids: A (adenine), C (cytosine), G (guanine) and T (thymine), wherein A and T, G and C are complementary pairs. Binary is a number system widely used in computing technology. Since binary data is represented by two complementary numbers of 0 and 1, so 00 and 11, 01 and 10 are also complementary. Therefore, binary arrays can be represented by complementary base pairs. According to the principle of base complementary pairing, only 8 coding rules meet Watson-Crick supplementary rule [25] as shown in Table 1.

**Table 1:** DNA encoding rules.

Rule	1	2	3	4	5	6	7	8
A	00	00	11	11	01	10	01	10
G	11	11	00	00	10	01	10	01
C	10	01	10	01	00	00	11	11
T	01	10	01	10	11	11	00	00

Taking the grayscale value “167” of image as an example, its binary sequence is “10100111”, and sequence “AAGT” is obtained by using DNA encoding rule 6; if the sequence is “AAGT” using DNA decoding rule 4 for decoding, the corresponding binary sequence is “11110010” and that converted to decimal system is “242”. Therefore, it can be efficient and convenient to achieve a change in pixel values only by DNA encoding.

As the research of DNA encryption algorithm becomes more and more perfect, operations between DNA sequences arise at the historic moment. DNA manipulation is based on the rules of binary arithmetic. The XOR operation of DNA coding [26] is shown in Table 2. According to an example given in Table 2, the result of XOR of DNA sequence “ACGT” and “CGAT” is “CTGA”.

**Table 2:** DNA XOR operation.

XOR	A	T	C	G
A	A	T	C	G
T	T	A	G	C
C	C	G	A	T
G	G	C	T	A

## 3. Double scrambling-DNA row and column closed loop dynamic encryption algorithm

**Fig 6:**
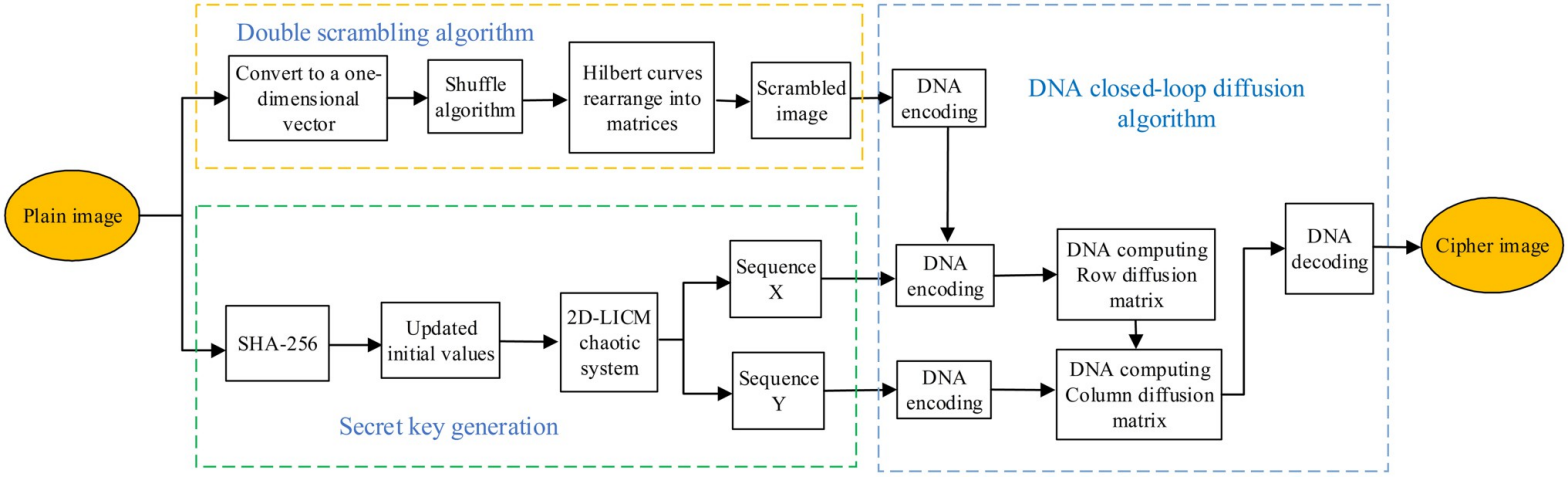
The encryption flow diagram of the proposed scheme.

The architecture of the cryptosystem is shown in Fig 6, including three stages: pixel scrambling stage, diffusion stage and key stream generation stage. Pixel reconstruction is implemented by double scrambling to ensure the confusion. In the first round, the pixels of the image are arranged as one-dimensional vectors, which are scrambled by Knuth-Durstenfeld shuffle algorithm. In the second round, the one-dimensional vectors are rearranged into matrices by using Hilbert curve to achieve another scrambling. The scrambled image and chaotic sequences *X* and *Y* are encoded by DNA, and the scrambled image rows and columns are encrypted by using closed-loop dynamic update diffusion method.

### 3.1 Secret key generation

Since the hash 256 is a single-item hash function, it has many excellent characteristics, such as a very fast hash value calculation, a fixed-length output, one-way irreversible and sensitive initial value, etc. In this paper, we will use hash 256 function to calculate the initial value of a two-dimensional cascade modulation coupled chaotic system (2D-LICM). Since the input of hash 256 function is the pixel value of the initial grayscale image, different hash sequences will be generated if the initial image is slightly different, so the calculated chaotic initial values will be different, which greatly increases the key space and calculation sensitivity of this paper. The 256 bits key is divided into 32 groups with 8 bits in each group, which can be expressed as

$$\begin{array}{c} \begin{array}{c} K=k_{1},k_{2},...,k_{32},\\ k_{i}=\{k_{i,0},k_{i,1},...,k_{i,7}\} \end{array} \end{array}$$ (2)

where in *k*_*i*,*j*_, *i* is the number of character and *j* is the number of bits. The initial value of the 2D-LICM chaotic system can be expressed as

$$\begin{array}{c} \left\{ \begin{array}{cc} & S1=k_{1}⊕k_{5}⊕k_{9}⊕k_{13}⊕k_{17}⊕k_{21}⊕k_{25}⊕k_{29}\\ & S2=k_{2}⊕k_{6}⊕k_{10}⊕k_{14}⊕k_{18}⊕k_{22}⊕k_{26}⊕k_{30}\\ & S3=k_{3}⊕k_{7}⊕k_{11}⊕k_{15}⊕k_{19}⊕k_{23}⊕k_{27}⊕k_{31}\\ & S4=k_{4}⊕k_{8}⊕k_{12}⊕k_{16}⊕k_{20}⊕k_{24}⊕k_{28}⊕k_{32} \end{array}\right. \end{array}$$ (3)

$$\begin{array}{c} \left\{ \begin{array}{cc} & x_{0}=(S1+S4)/256\\ & y_{0}=(S2+S4)/256 \end{array}\right. \end{array}$$ (4)

where *x*_0_ and *y*_0_ are the initial values of two-dimensional cascade modulation coupled chaotic system (2D-LICM) and *x* ⊕ *y* is the XOR operation of *x* and *y*. Iterate the chaotic system 4 × *M* × *N* times and get two chaotic sequences *X* and *Y* with the length of 4 × *M* × *N*, where *X* = [*x*_1_, *x*_2_, …, *x*_4*MN*_] and *Y* = [*y*_1_, *y*_2_, …, *y*_4*MN*_].

### 3.2 Double scrambling algorithm

In the process of image scrambling, Hilbert curve is usually applied to scan the pixel points of the image matrix according to the traversal rules of the curve, store them in a one-dimensional sequence, and then rearrange the image pixel points to generate scrambled images. However, because the coding method of Hilbert curve will store the adjacent objects together in space, it greatly reduces the scrambling effect of image pixels. This paper will combine the Hilbert curve with the Knuth-Durstenfeld shuffle algorithm, and Hilbert curve is applied to rearrange one-dimensional vector into matrix according to the coding mode of curve, which implements double scrambling of image, improves the efficiency of data processing in memory, and has better scrambling effect in the encryption process.

Suppose the size of gray image P is *M* × *N*. The encryption algorithm of scrambling method is shown in Algorithm 1:

**Algorithm 1** Double scrambling method

**Input:** Plain image *P*.

**Output:** Scrambled image *PH*.

1: [*M*, *N*] ← size of plain image.

2: *U*1 = *reshape* (*P*, 1, *M* * *N*) ← Plain image *P* is arranged as one-dimensional vector.

3: *n* = *length*(*U*1)

4: **for** *i* = *n*: −1: 2 **do**

5:  *r* = *floor* (*rand* *) + 1

6:  *t* = *P*(*r*)

7:  *P*(*r*) = *P*(*i*)

8:  *P*(*i*) = *t*

9: **end for**

10: *PH* = *Hilbert*(*P*(*r*)) ← Rearrange *P*(*r*) into matrix by Hilbert curves.

**Fig 7:**
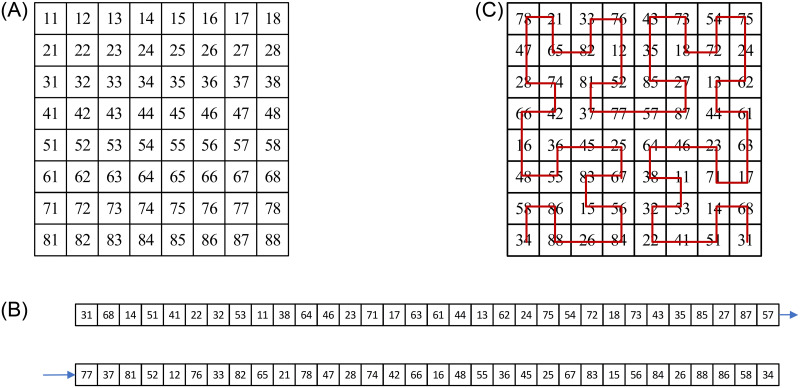
Numerical example of double scrambling method. A: Original pixel matrix. B: Matrix with shuffling algorithm. C: The reconstructed matrix with Hilbert curve.

In order to better explain the image scrambling effect, Fig 7 shows a numerical example of the double scrambling method. Fig 7(A) shows the original pixel matrix, Fig 7(B) shows the pixel position after shuffling the pixel matrix as one-dimensional vector, and Fig 7(C) shows the pixel matrix by arranging the one-dimensional vectors from the lower right corner according to the Hilbert curve coding form. Through the above two scrambling stages, the correlation between adjacent pixels in the original image can be effectively reduced.

### 3.3 Row and column closed-loop diffusion algorithm

In the process of image encryption and transmission, it is usually block transmission, but it has a high requirement on the size of the image. In order to improve the rate of encryption and transmission, the diffusion operation proposed in this paper is based on DNA coding row and column closed-loop dynamic update operation. In this algorithm, we improve the existing block closed-loop diffusion scheme, using the two-round diffusion of row and column to encode the scrambled image with DNA. Due to the tremendous parallelism, high storage density and complex encoding method of DNA, the problems of low efficiency and poor security in the traditional encryption process are solved. In the process of encryption, the secret key is composed of the random mask and the previous line ciphertext, which increases the interrelation between the plaintext, ciphertext and the key stream. In this paper, the last line ciphertext is used to update the first line, and realizes the dynamic closed-loop diffusion operation. The implementation of the algorithm is mainly divided into two phases.

***PhaseI***: Row and column encryption algorithm.

The pixel diffusion operation of the scrambled image with the size of *M* * *N* is described as follows.

Step 1: The scrambled image *PH*, chaotic sequence *X* and *Y* are converted into binary, and DNA coding operation is performed on them to obtain the matrices *PH*1, *X*1 and *Y*1, which are M * 4N in size.
Step 2: The first line of the ciphertext is obtained by the XOR operation on the first line of the plaintext and the first line of the matrix *X*1. The calculation formula is as follows:
  $$\begin{array}{c} \begin{array}{c} XOR\_1\{1,i\}(p,q)=XOR(\left(PH1\{1,i\}(p,q),X1\{1,i\}(p,q)\right) \end{array} \end{array}$$ (5)
  where {1, *i*} denote all the elements in the first row, *i* represents the *i* − *th* column of the matrix, *PH*1{1, *i*}(*p*, *q*) represents the pixel value of the pixel (*p*, *q*) in the first row of the scrambled image after DNA coding, and *X*1{1, *i*}(*p*, *q*) represents the pixel value of (*p*, *q*) in the first row of chaotic sequence *X*1 after DNA coding.
Step 3: The first line of the ciphertext is used as the key of the next line encryption, and the ciphertext of the second line is obtained by XOR of plaintext of the second line, the second line of matrix X1 and the first line of ciphertext. The calculation formula is as follows:
  $$\begin{array}{c} XOR\_temp=XOR\_1\{1,i\} \end{array}$$ (6)
  $$\begin{array}{c} \begin{array}{c} XOR\_1\{2,i\}(p,q)=XOR\left(PH1\{2,i\}(p,q),X1\{2,i\}(p,q)\right) \end{array} \end{array}$$ (7)
  $$\begin{array}{c} \begin{array}{c} XOR\_2\{2,i\}(p,q)=XOR(\left(XOR\_1\{2,i\}(p,q),XOR\_temp(p,q)\right) \end{array} \end{array}$$ (8)
  where *XOR*_*temp* represents the key to encrypt the next line.
Step 4: Update the ciphertext value as the key to encrypt the next line.
  $$\begin{array}{c} XOR\_temp=XOR\_2\{2,i\} \end{array}$$ (9)
Step 5: Repeat steps 3 and 4 until all rows are encrypted.

***PhaseII***: Closed-loop update algorithm.

Step 1: The encrypted image is decoded by DNA, and the calculation formula for the encrypted image is as follows:
  $$\begin{array}{c} PH2\{1,:\}(p,q)=DNA\_decrypt{\left(XOR\_1\right)}^{\prime } \end{array}$$ (10)
  $$\begin{array}{c} PH2\{i,:\}(p,q)=DNA\_decrypt{\left(XOR\_2\right)}^{\prime } \end{array}$$ (11)
Step 2: Update the first line of ciphertext image with the last line to realize closed-loop diffusion operation. The update method is as follows:
  $$\begin{array}{c} C\{1,:\}(p,q)=XOR\left(PH2\{1,:\}(p,q),PH2\{i,:\}(p,q)\right) \end{array}$$ (12)
Step 3: After the ciphertext of all rows are generated, in order to ensure the security of the encrypted image, this method will continue to be used to encrypt the columns of the image.
Step 4: A complete ciphertext image is obtained by integrating the updated ciphertext of row and column, which is the final ciphertext image *C*.

The above steps are the row-column closed-loop dynamic update diffusion method based on DNA coding, and the algorithm code is shown in Algorithm 2 and Algorithm 3.

**Algorithm 2** Row-column encryption algorithm.

**Input:** Scrambled image *PH*1, the chaotic sequence *X*..

**Output:** The encrypted image *PH*2.

1: [*M*, *N*] ← size of scrambled image.

2: *X*(1:*M* × *N*) ← Convert *X* to one-dimension array.

3: **for** *i* ← to *M* **do**

4:  *PH*1_*encode* = *DNA*_*encrypt*(*PH*1(*i*,:)) ← code the scrambled image using DNA

5:  *X*_*encode* = *DNA*_*encrypt*(*X*(*i*,:)) ← code chaotic sequences using DNA

6: **end for**

7: **for** *k* ← to *N* **do**

8:  *XOR*_1(*k*, *j*) = *DNA*_*XOR*(*PH*1_*encode*(*k*, *j*), *X*_*encode*(*k*, *j*)) ← Scrambled image and chaotic sequences performing DNA operation

9:  **if** *i* ∼ = 1 **then**

10:   *XOR*_2(*k*, *j*) = *DNA*_*XOR*(*XOR*_1(*k*, *j*), *XOR*_*temp*(*k*, *j*))

11:  **end if**

12: **end for**

13: **if** *i* = 1 **then**

14:  *XOR*_*temp* = *XOR*_1

15: **else**

16:  *XOR*_*temp* = *XOR*_2

17: **end if**

**Algorithm 3** Closed-loop update algorithm.

**Input:** The encrypted image *PH*2

**Output:** The final encrypted image *C*.

1: *PH*2_*encrypt*(1,:) = *DNA*_*decrypt*(*XOR*_1) ← The first row of matrix performs DNA decoding

2: *PH*2_*encrypt*(*i*,:) = *DNA*_*decrypt*(*XOR*_2) ← The other rows of matrix performs DNA decoding

3: *C*(1,:) = *XOR*(*PH*2_*encrypt*(1,:), *PH*2_*encrypt*(*i*,:)) ← Update the first row of matrix

4: **return** *C*

**Fig 8:**
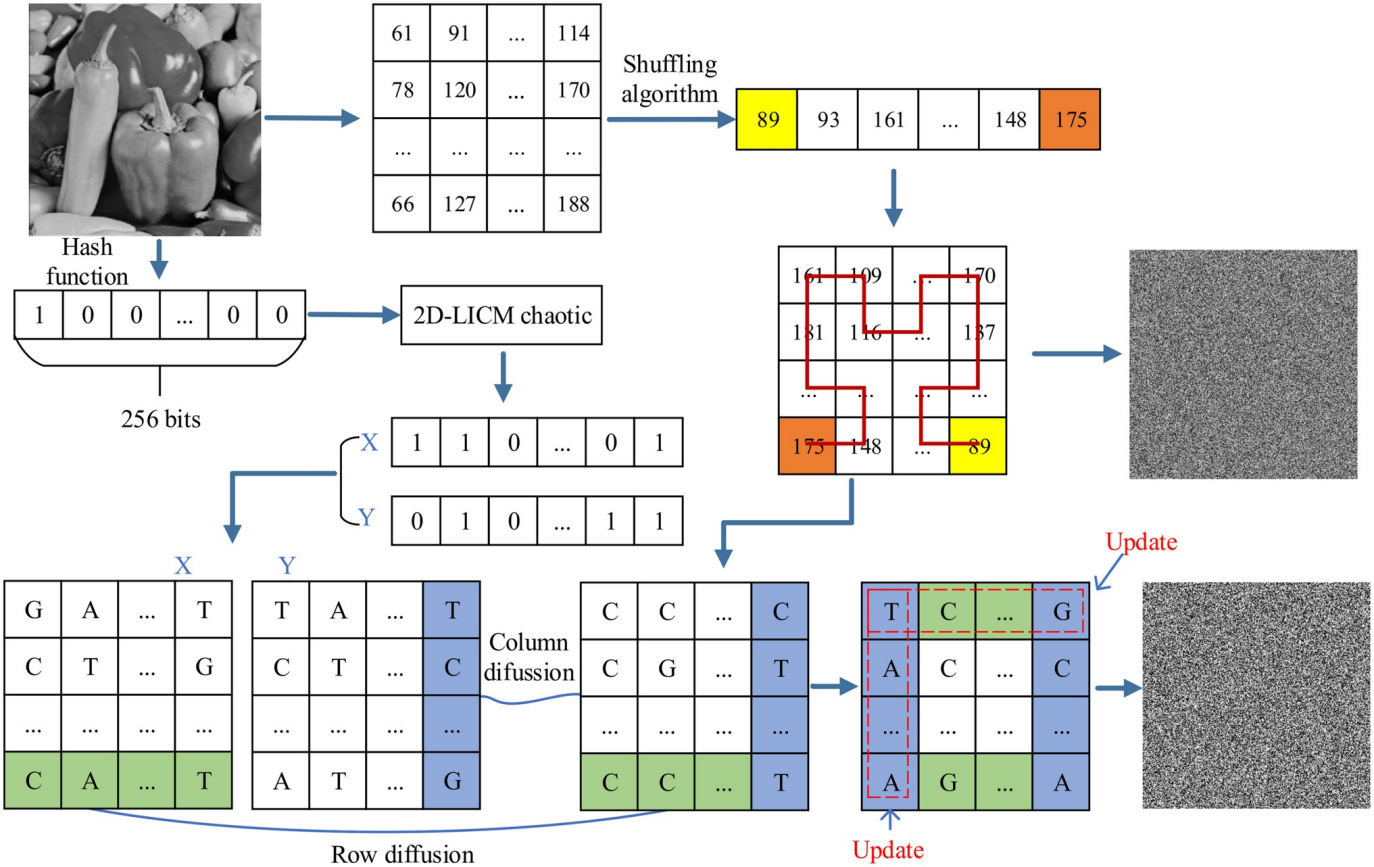
Flowchart of Peppers encryption step (size 256 x 256).

Taking the grayscale Peppers diagram with a size of 256 × 256 as an example, the encryption step flowchart is shown in Fig 8. From the process of closed-loop dynamic update diffusion method based on DNA coding, we can see some highlights of encryption scheme. First, because DNA has vast parallelism, extraordinary storage density, the security and encryption effect of this paper are improved. Secondly, the parameter of the two-dimensional cascade modulation coupled chaotic system (2D-LICM) is obtained by SHA-256 of the original image, and the random sequence is formed by a certain number of iterations. In addition, each ciphertext line is constructed by the current plaintext line, the key line and the ciphertext line generated by the uplink. Therefore, the combination of plaintext, ciphertext, key stream, and DNA can better prove the security of the encryption system.

## 4. Simulation experiment and security analysis

**Fig 9:**
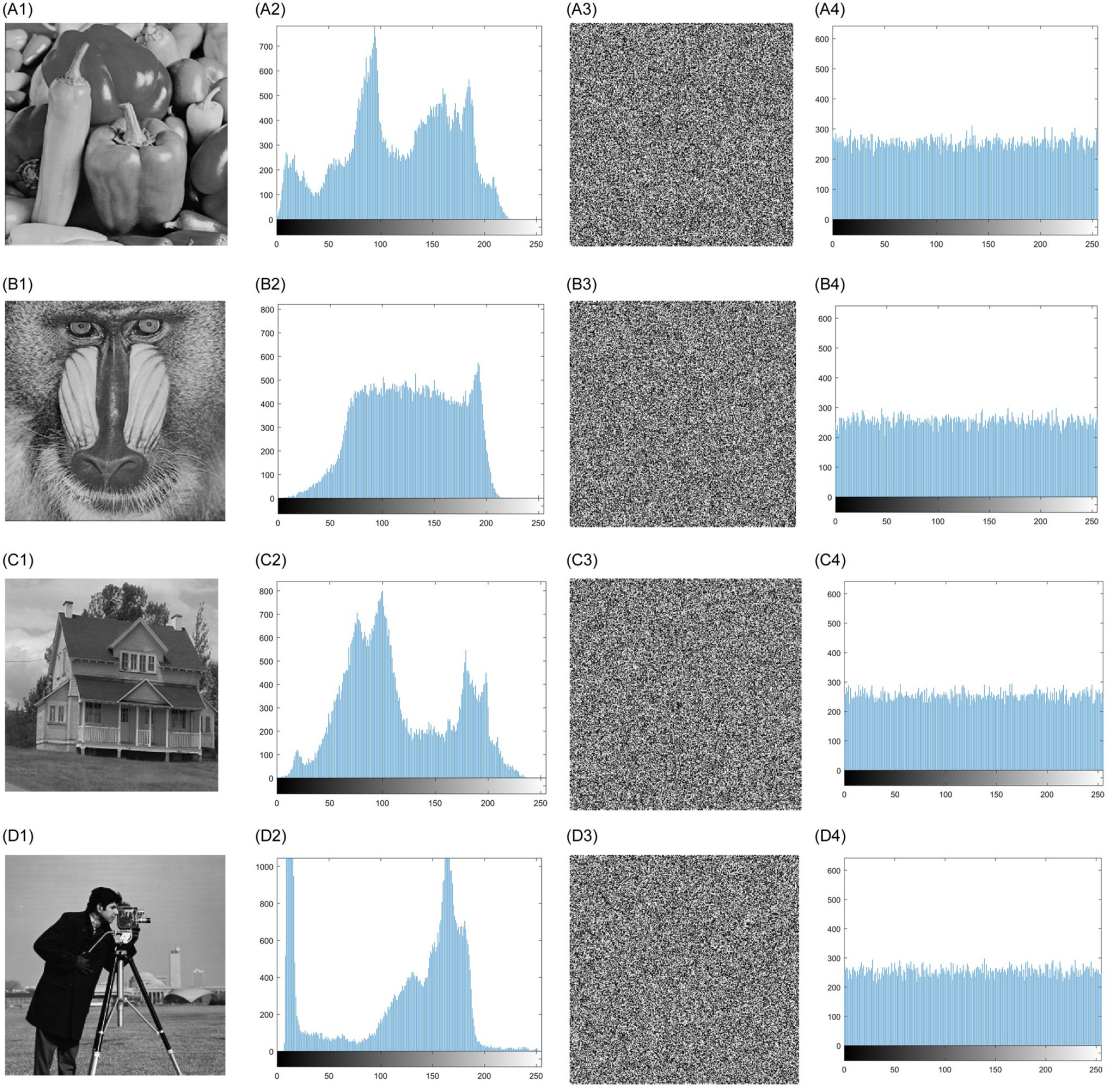
Histogram analysis of original and encrypted images. A1-D1: Original images of Peppers, Baboon, House and Cameraman. A2-D2: The corresponding original image histograms. A3-D3: The encrypted images of Peppers, Baboon, House and Cameraman. A4-D4: The corresponding encrypted image histograms.

In this experiment, MATLAB 2018b is used to simulate and evaluate the proposed algorithm. In order to prove the feasibility and efficiency of the algorithm, we have performed many experiments on general image sets and representative experimental images. In Fig 9, four images of Peppers, Baboon, House and Cameraman with the size of 256 × 256 are selected as test images. The following sections will discuss the experimental effect and security analysis and compare the proposed with similar algorithms.

### 4.1 Histogram analysis

Histogram analysis needs to count the data of samples firstly and display the distribution state of each data by using two-dimensional statistical table. Its coordinates are, respectively, the grayscale image level and the number or probability of the corresponding pixel appearing in the image. Histogram analysis is a meaningful basis to evaluate the statistical performance of image encryption algorithms. The more stable its distribution is, the lower the identifiability of ciphertext image is and the higher its ability to resist statistical attacks is.

Fig 9(A1)–9(D1) show the input original image of Peppers, Baboon, House and Cameraman, Fig 9(A2)–9(D2) show the corresponding original image histograms, Fig 9(A3)–9(D3) show the secret image of Peppers, Baboon, House and Cameraman,and Fig 9(A4)–99(D4) show the histogram of the corresponding secret images. Taking the gray-scale Peppers image as an example, it can be seen from that the distribution of the plaintext image histogram has obvious volatility and significant regularity, while the grayscale of the ciphertext image histogram is uniform distributed, and the pixel value distribution cannot be recognized by humans, which proves that the algorithm in this paper has good encryption performance.

### 4.2 Key space analysis

The key length of a secure image encryption algorithm should be long enough. Generally speaking, the larger the length of the key is, the more difficult it is for the attacker to guess the password exhaustively. Initial conditions for several variables of two-dimensional cascade modulation coupled chaotic system (2D-LICM) have certain limits, where *a* ∈ (0, ∞), *k* ∈ (0, ∞), *x*_0_ and *y*_0_ are determined by parameters *S*1, *S*2, *S*3 and *S*4, as well as the section of DNA coding, decoding and operation rules. If the calculation accuracy of the computer is 10^−15^, the key space is:

$$\begin{array}{c} 10^{-15}\times 10^{-15}\times 10^{-15}\times 10^{-15}\times 10^{-15}\times 10^{-15}\times 10^{-15}\times 10^{-15}\times 10^{-15}=10^{-135}>2^{-445} \end{array}$$ (13)

Table 3 shows the key space comparison of multiple encryption algorithms, and it can be concluded that the key space of the proposed algorithm is long enough to meet the actual security requirements.

**Table 3:** Comparison of key spaces.

Algorithm	Ref [27]	Ref [28]	Ref [29]	Ref [30]	Proposed
Key space	2^193^	2^189^	2^190^	2^256^	2^445^

### 4.3 Correlation coefficient analysis

**Fig 10:**
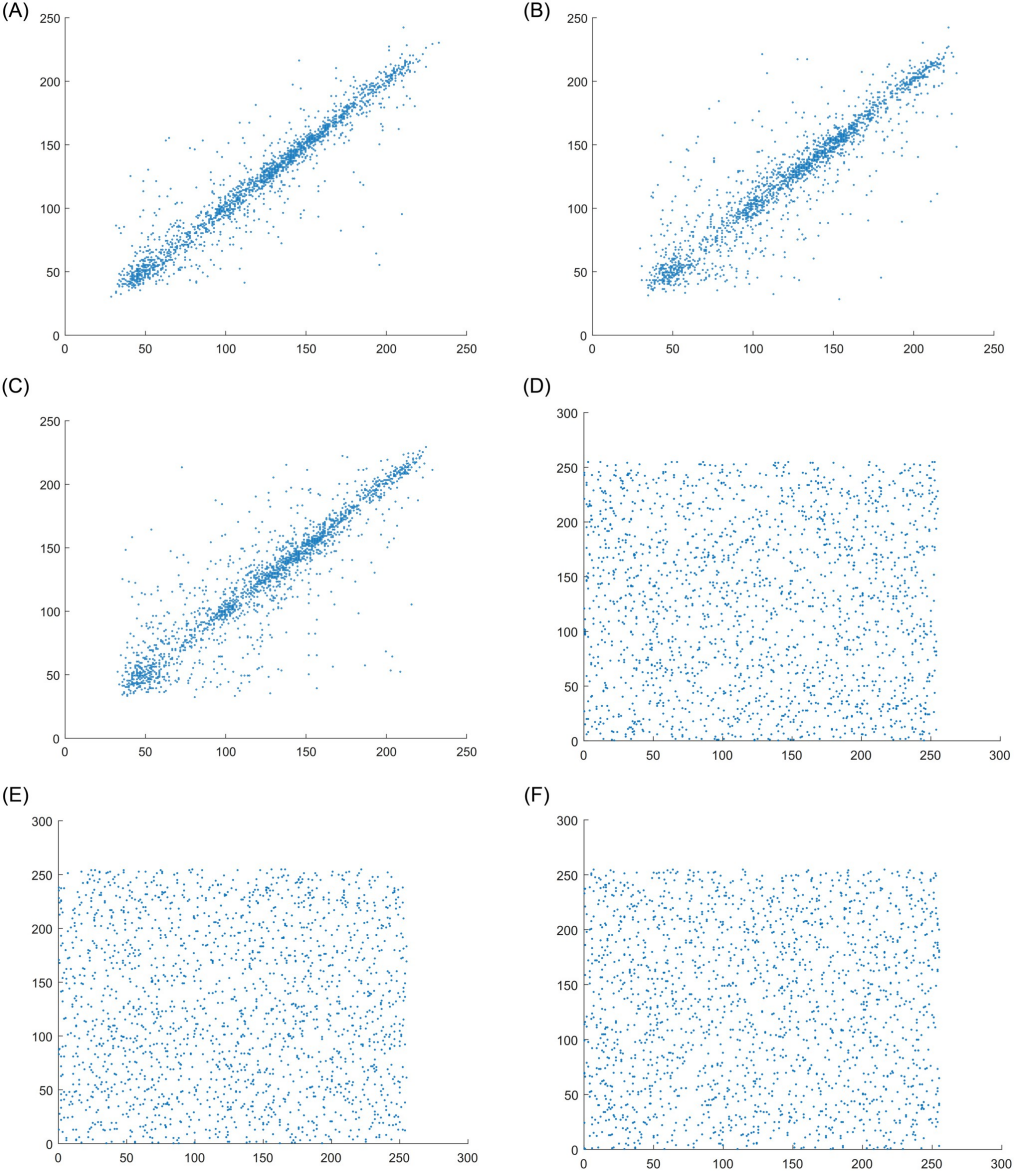
Pixel correlation analysis of plain image Peppers and corresponding encrypted image. A: Horizontal pixel of plain image. B: Vertical pixel of plain image. C: Diagonal pixel of plain image. D:Horizontal pixel of cipher image. E:Vertical pixel of cipher image. F:Diagonal pixel of cipher image.

There exists a highly correlation between the adjacent pixels of original image. Image encryption technology is mainly used to break the correlation between pixels. Researchers often use the correlation coefficient of probability theory to measure the quality of the encryption effect. The mathematical formula used in the correlation analysis is as follows:

$$\begin{array}{c} E(x)=\frac{1}{N}\sum_{i=1}^{N}x_{i} \end{array}$$ (14)

$$\begin{array}{c} D(x)=\frac{1}{N}\sum_{i=1}^{N}{\left(x_{i}-E(x)\right)}^{2} \end{array}$$ (15)

$$\begin{array}{c} \text{cov}(x,y)=\frac{1}{N}(x_{i}-E(x))(y_{i}-E(y)) \end{array}$$ (16)

$$\begin{array}{c} r_{xy}=\frac{\text{cov}(x,y)}{\sqrt{D(x)}\sqrt{D(y)}} \end{array}$$ (17)

where *x* and *y*, respectively, represent the grayscale of adjacent pixels, *N* is sum of all pixels, *E*(*x*) and *D*(*x*) are the expectation and variance of variable *x*, *E*(*y*) and *D*(*y*) are the expectation and variance of variable *y*, cov(*x*, *y*) is the covariance of variable *x* and *y*, and *r*_*xy*_ is the correlation between two adjacent pixels. Select 3000 pixels from the horizontal, vertical and diagonal directions of the ciphertext image and analyse the correlation between them. It can be seen from the Table 4 that the correlation coefficient in each direction before the encrypted image is high and very close to 1. After the encrypted image, the correlation coefficient is close to 0 and the correlation is low. It can be seen from Fig 10 that the encrypted image has uniform distribution characteristics. Good scrambling and diffusion methods can effectively weaken the correlation between adjacent pixels.

**Table 4:** Adjacent pixel correlation analysis of Peppers image.

Direction	proposed	Ref [30]	Ref [31]	Ref [32]
Horizontal	**0.0039**	0.0066	0.0068	-0.0036
Vertical	**0.0174**	0.0261	-0.0054	0.0023
Diagonal	**−0.0034**	0.0134	0.0010	0.0022

### 4.4 Local and global entropy analysis

In many image encryption processes, it is necessary to judge the clarity of an image, and information entropy is used as a quantitative standard to evaluate the randomness of information. Its main function is to evaluate the uncertainty and unpredictability of the system. The general formula for calculating information entropy is as follows:

$$\begin{array}{c} H(x)=-\sum_{i=0}^{n-1}p(x_{i}){\text{log}}_{2}p(x_{i}) \end{array}$$ (18)

where *n* is the total number of pixels, *p*(*x*_*i*_) is the probability of grayscale *x*_*i*_. In theory, the closer the numerical value of information entropy is to 8, its information will greatly reduce the likelihood of leaks. It can be seen from Table 5 that the range of the information entropy of the original image is between 6.3908 and 7.4874, but the information entropy after the encryption scheme can reach more than 7.9970, with an average value of 7.9981. Compared with literature [33], the information entropy value of the image encrypted by the proposed scheme is larger, and the information entropy of the encrypted image is closer to 8, which means that the encrypted image is closer to the random source, so the proposed encryption algorithm can resist information entropy analysis attacks.

**Table 5:** Analysis and comparison of global entropy and local entropy between original image and encrypted image.

Test Image	Global entropy			Local entropy (*k*, *T*_*B*_, *α*) = (30, 1936, 0.001)		
	Plain image	Ref [33]	Proposed	Plain image	Ref [33]	Proposed
Baboon	7.3715	7.9971	7.9973	6.7807	**7.9021**	**7.9028**
Barbara	6.5838	7.9973	7.9984	6.7223	7.9014	**7.9027**
Boat	7.1612	7.9971	7.9993	6.3282	**7.9029**	7.9035
Couple	6.1689	7.9970	7.9970	6.3158	7.8987	**7.9020**
Chemical plant	7.0193	7.9973	7.9982	6.6642	7.9002	**7.9025**
Clock	7.2943	7.9954	7.9989	6.4612	**7.9023**	**7.9022**
Elaine	7.4874	7.9971	7.9980	6.4139	**7.9020**	**7.9025**
Fingerprint	6.5945	7.9971	7.9979	7.2007	**7.9029**	**7.9024**
Gold Hill	7.4460	7.9975	7.9981	6.4601	**7.9020**	**7.9026**
Peppers	7.3797	7.9970	7.9974	6.4479	7.9043	**7.9024**
Plane	6.3908	7.9973	7.9991	6.1305	**7.9016**	7.9037
Resolution chat	7.4590	7.9963	7.9971	7.0599	7.9035	**7.9028**
MEAN	7.0297	7.9970	7.9981	6.5821	**7.9020**	**7.9027**
PASS/ALL	-	-	-	-	7/12	10/12

The calculation of local information entropy [34] divides the original image into multiple nonoverlapping small blocks and randomly selects the sample mean of entropy of image blocks. The local information entropy is more random and faster in calculation than the global information entropy and allows fair comparison between images of different sizes. Its formula is

$$\begin{array}{c} H_{k.T_{B}}(S)=\sum_{i=1}^{k}\frac{H(S_{i})}{k} \end{array}$$ (19)

where *S* represents the original image, *k* and *T*_*B*_ represent the number of blocks and pixels of each block respectively, and *H*(*S*_*i*_) represents the global information entropy of block *S*_*i*_. According to the central limit theorem, when *k* > 30, the sample mean of the local information entropy is approximately a normal distribution. In this experiment, we select *k* = 30 and *T*_*B*_ = 1936, take the significance level *α* as 0.001, Therefore, we can get the pass interval of the local information entropy of the encrypted image as [7.901515698, 7.903422936]. The test results are shown in Table 5. In the local information entropy test, the pass rate of the proposed algorithm is 10/12. In the literature [33], the pass rate of the proposed algorithm is 7/12, and the average value of the local information entropy of the test image is also within this interval, which indicates that the algorithm has high security.

### 4.5 Known plaintext attack and chosen plaintext attack

**Fig 11:**
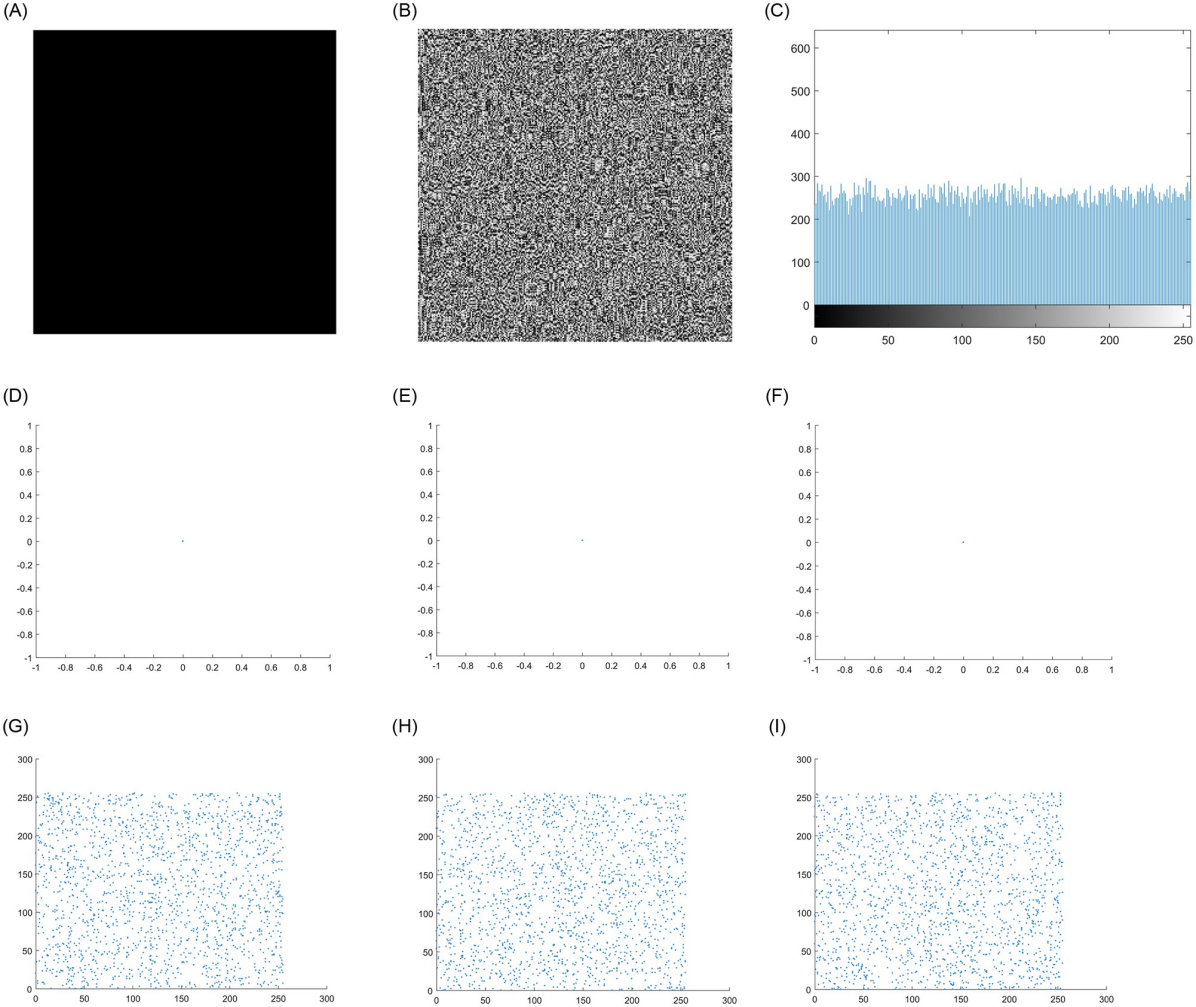
Analysis of encryption test results of all black images. A: Original full black image. B: Encrypted full black image. C: Histogram of encrypted image. D: Horizontal pixel of original image. E: Vertical pixel of original image. F: Diagonal pixel of original image. G: Horizontal pixel of encrypted image. H: Vertical pixel of encrypted image. I: Diagonal pixel of encrypted image.

**Fig 12:**
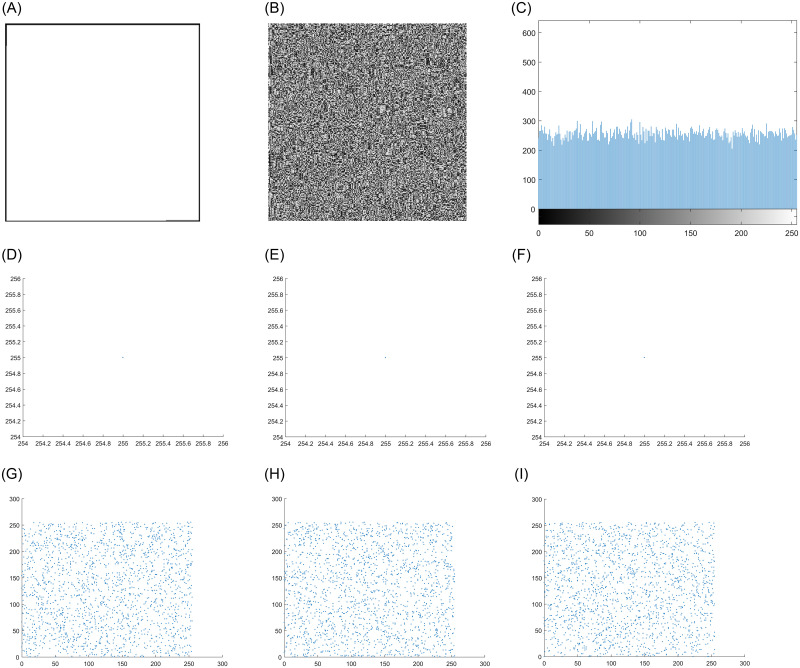
Analysis of encryption test results of all white images. A: Original full white image. B: Encrypted full white image. C: Histogram pixel of encrypted image. D: Horizontal pixel of original image. E: Vertical pixel of original image. F: Diagonal pixel of original image. G: Horizontal pixel of encrypted image. H: Vertical pixel of encrypted image. I: Diagonal pixel of encrypted image.

There are four typical attacks in the field of cryptanalysis, of which the chosen-plaintext attack is the most powerful attack. It refers to certain information data in the encrypted image that the attacker knows in advance. Due to the inherent nonlinearity of DNA operation, the input and output of encryption system are not a simple linear relationship. However, this algorithm can apply dynamic DNA coding rules to all pixels of ordinary images to realize image encryption. In some cases, attackers will choose some special types of original images to prove the insecurity of the system. In this case, we encrypt all white and all black images to test the performance of the system against these powerful attacks. Figs 11 and 12 show the test results. It can be clearly obtained from the figure that the encrypted image is similar to the noise, and the histogram and pixel distribution of image are completely different from the original image. It can be seen that the system can effectively prevent known plaintext attack and chosen plaintext attack. Table 6 shows the correlation coefficient analysis and entropy analysis of all black and white images.

**Table 6:** Black and white correlation coefficient and entropy.

Image	Entropies	Correlation coefficients		
		Horizontal	Vertical	Diagonal
Full black	0	-	-	-
Cipher image of black	7.9970	0.0211	-0.0125	-0.0134
Full white	0	-	-	-
Cipher image of white	7.9972	-0.0306	0.0057	-0.0247

### 4.6 NPCR and UACI

Differential attack is a method of attacking and deciphering image information, mainly to make minor changes to the original image data. In order to effectively resist differential attacks, we use encryption algorithms to separately encrypt the changed image and the original image. When the two encrypted images show great differences, it shows that the encryption algorithm is very sensitive. To resist differential attack, the two indexes of pixel number change rate (NPCR) and uniform mean intensity of change (UACI) are often used to perform quantitative and qualitative analysis on the processed images. The literature [35] provides strict criteria for determining whether an image can pass the NPCB and UACI tests. The calculated theoretical values of NPCR and UACI were 99.6094 and 33.4635, respectively. Their calculation formula is as follows:

$$\begin{array}{c} D(i,j)=\{\begin{array}{cc} & 0,c_{1}(i,j)=c_{2}(i,j)\\ & 1,c_{1}(i,j)\neq c_{2}(i,j) \end{array} \end{array}$$ (20)

$$\begin{array}{c} NPCR=\frac{\sum_{i=1}^{M}\sum_{j=1}^{N}D(i,j)}{M\times N}\times 100 \end{array}$$ (21)

$$\begin{array}{c} UACI=\frac{1}{M\times N}\sum_{i=0}^{M}\sum_{j=0}^{N}\frac{\left|c_{1}(i,j)-c_{2}(i,j)\right|}{255}\times 100 \end{array}$$ (22)

where *c*_1_(*i*, *j*) and *c*_2_(*i*, *j*) represent the two pixel values of (*i*, *j*) at the same position in two different encrypted images, *M* and *N* are the dimension of the image. Since the initial value of the chaos is calculated by the hash function, a change in the value of a pixel in the plaintext image will result in a substantial change in the chaotic sequence. As shown in Table 7, we test Baboon, Peppers and Plane, and compare the results with other algorithms. Each image is tested 50 times, and the average value is recorded. From the data, it can be seen that the algorithms NPCR and UACI proposed in this paper are very close to the theoretical values, therefore it is verified that the proposed image encryption scheme can resist differential attack.

**Table 7:** Comparison of NPCR and UACI.

Image	NPCR				UACI			
	Proposed	Ref [25]	Ref [26]	Ref [30]	Proposed	Ref [25]	Ref [26]	Ref [30]
Baboon	99.6353	96.8450	99.6143	99.6348	33.4898	32.4069	33.4675	33.4725
Peppers	99.6387	99.0281	99.6135	99.6348	33.5359	33.1006	33.4692	33.3637
Plane	99.6248	98.5591	99.6226	99.5855	33.5068	33.1368	33.4251	33.5052

### 4.7 MSE, PSNR and MAE

Mean square error (MSE), peak signal-to-noise ratio (PSNR) and mean absolute error (MAE) are often used as important indexes to measure image robustness. The mathematical expressions of MSE, MAE and PSNR are as follows:

$$\begin{array}{c} MSE=\frac{1}{M\times N}{\sum_{i=1}^{M}\sum_{j=1}^{N}[P(i,j)-C(i,j)]}^{2} \end{array}$$ (23)

$$\begin{array}{c} PSNR=10\times {\text{log}}_{10}(\frac{255^{2}}{MSE}) \end{array}$$ (24)

$$\begin{array}{c} MAE=\frac{1}{M\times N}\sum_{i=1}^{M}\sum_{j=1}^{N}|P(i,j)-C(i,j)| \end{array}$$ (25)

where *P* and *C* are plaintext images and ciphertext images respectively, (*i*, *j*) is pixel position of the image, and *M* and *N* refer to the image size. PSNR is the most common and widely used objective index of image evaluation, and it is based on the error of corresponding pixel points. Because the visual characteristics of human eyes are not taken into account, the evaluation results are often inconsistent with people’s subjective feelings. It can be seen from Eq (24) that there exists an inversely proportional relationship between PSNR and MSE. At present, the mean absolute error is also widely used in the analysis of the difference between the test images. In the test, we changed the one-bit pixel value of the plaintext image and also tested at the same time. According to the analysis of the results in Table 8, the smaller the PSNR measured by the algorithm for all test images, the larger the MSE. Therefore, there is a great difference between the plaintext image and the ciphertext image, and the algorithm has better encryption effect.

**Table 8:** Comparison of MAE, MSE and PSNR.

Image	Plain-encrypted images			After one bit changed in plain image		
	MAE	MSE	PSNR	MAE	MSE	PSNR
Peppers	73.6438	7849.3	9.3681	73.4192	7815.1	9.3422
Baboon	71.3447	7343.9	9.4715	71.2780	7319.4	9.4860
Terrace	87.3575	11445.6	7.5444	87.2861	77414.9	7.5561
Plane	83.8329	10089.3	7.3979	83.6259	10076.2	7.3983
Cameramen	79.5888	9422.0	8.3894	79.5472	9438.5	8.3818
Couple	84.2192	9462.3	7.8686	84.2471	9463.9	7.8616

### 4.8 Key sensitivity analysis

Key sensitivity is another basic feature of ideal encryption algorithm, which means that if the key is very slightly different, an entirely different encrypted image will be generated, and the key of this algorithm is composed of hash algorithm and chaotic system. Due to the hash algorithm and the chaotic system are extremely sensitive to initial conditions and parameters, thus improved key sensitivity of the scheme. The extremely high key sensitivity guarantees the security of the encryption system to a large extent and prevents brute force cracking attacks. In order to evaluate the sensitivity of the key, this paper changes 1 bit of the sub-key, while the other keys remain unchanged for testing.

**Fig 13:**
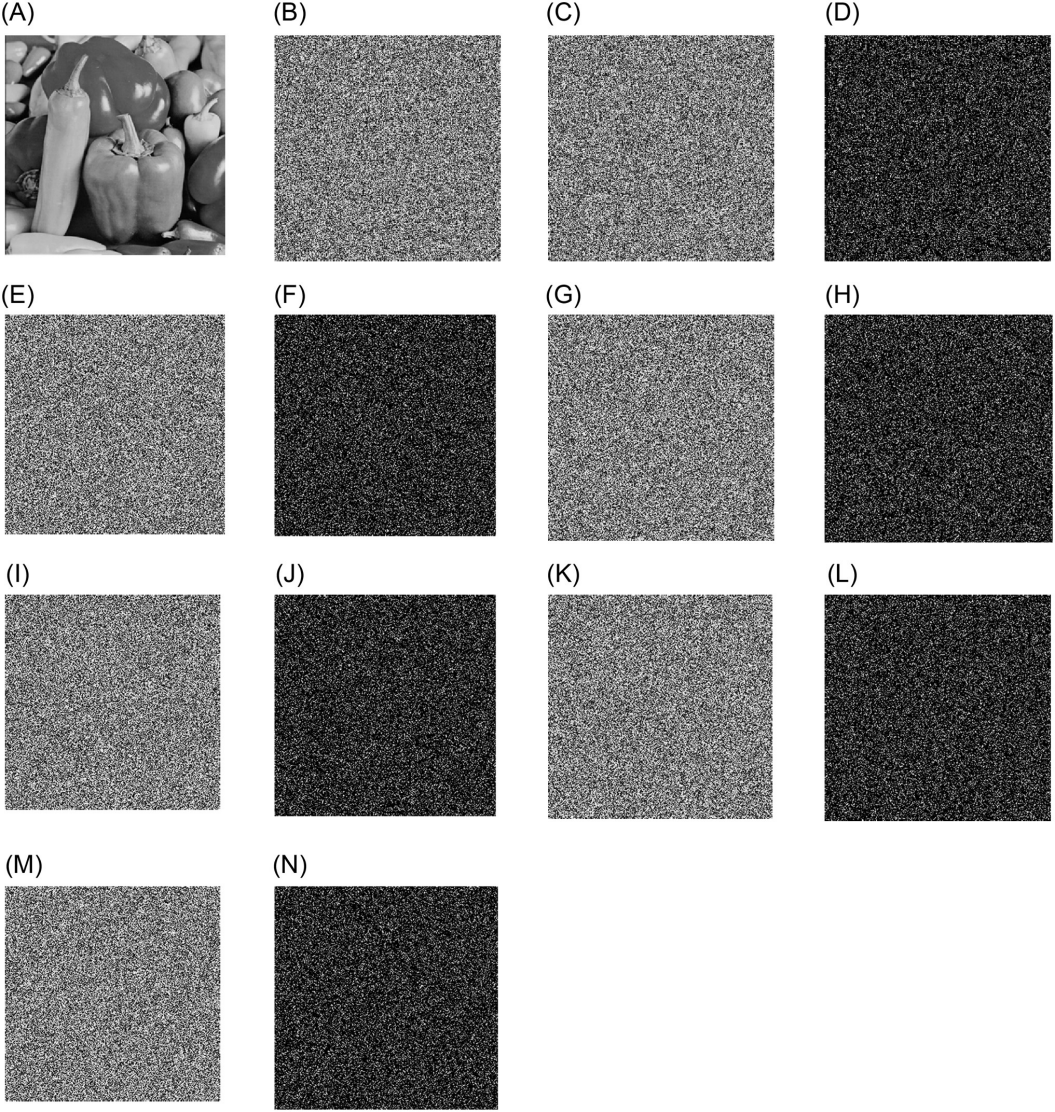
Key sensitivity analysis. A: Plain image of Peppers. B: Encrypted image *C* with correct key. C: Encrypted image *C*_1_ with key *S*1 changed by one bit. D: |*C*_1_ − *C*|. E: Encrypted image *C*_2_ with key *S*2 changed by one bit. F: |*C*_2_ − *C*|. G: Encrypted image *C*_3_ with key *S*3 changed by one bit. H: |*C*_3_ − *C*|. I: Encrypted image *C*_4_ with key *S*4 changed by one bit. J: |*C*_4_ − *C*|. K: Encrypted image *C*_5_ with key *x*_0_ changed by one bit. L: |*C*_5_−*C*|. M: Encrypted image *C*_6_ with key *y*_0_ changed by one bit. N: |*C*_6_ − *C*|.

Fig 13(A) shows plain image of Peppers, Fig 13(B) shows the process of encrypting the plain image with the correct key, and Fig 13(C) shows the re-encrypted image by changing the key *S*1 by one bit. In the case that the two encrypted images cannot be visually compared, this paper subtracts the two images. As can be seen from Fig 13(D), there are obvious differences between the two encrypted images. Fig 13(E) shows the re-encrypted image by changing the key *S*2 by one bit, Fig 13(F) is the difference image between Fig 13(B) and 13(E). Fig 13(G) is the re-encrypted image by changing the key *S*3 by one bit, Fig 13(H) is the difference image of Fig 13(B) and 13(G). Fig 13(I) is the re-encrypted image by changing the key *S*4 by one bit, and Fig 13(J) is the difference image between Fig 13(B), 13(I) and 13(K) is the re-encrypted image by changing the key *x*_0_ by one bit, Fig 13(L) is the difference image of Fig 13(B) and 13(K). Fig 13(M) is the re-encrypted image by changing the key *y*_0_ by one bit, and Fig 13(N) is the difference image of Fig 13(B) and 13(M). As can be seen from the figure, in the case of keeping other keys unchanged, changing only one bit of the key will result in two completely different encrypted images, which proves that our encryption algorithm is very sensitive to the key.

Additionally, to test the key sensitivity, while keeping other keys unchanged, we also analyze each subkey with 10^−13^ minor perturbations in Table 9. *key*_0_ and *key*_*i*_ have only one different key, the other keys are the same. It can be clearly seen from Table 9 that the difference rate between encrypted images is higher than 99.56%. This means that a small change in the key will cause a significant change in the encrypted image. Therefore, we can conclude that the encryption algorithm proposed in this study is extremely sensitive to keys.

**Table 9:** Difference rates between two images encrypted by slightly different keys.

Secret keys	Difference rates (%)					
	Peppers	Baboon	Terrace	Plane	Cameramen	Couple
*key*_1_(${x_{0}}^{{}^{\prime }}=x_{0}+10^{-13}$)	99.60	99.61	99.62	99.58	99.58	99.63
*key*_2_(${y_{0}}^{{}^{\prime }}=y_{0}+10^{-13}$)	99.59	99.62	99.58	99.61	99.57	99.61
*key*_3_(${S1}^{{}^{\prime }}=S1+10^{-13}$)	99.61	99.61	99.59	99.56	99.60	99.62
*key*_4_(${S2}^{{}^{\prime }}=S2+10^{-13}$)	99.63	99.59	99.60	99.60	99.61	99.63
*key*_5_(${S3}^{{}^{\prime }}=S3+10^{-13}$)	99.63	99.60	99.59	99.61	99.61	99.62
*key*_6_(${S4}^{{}^{\prime }}=S4+10^{-13}$)	99.62	99.61	99.61	99.58	99.60	99.59

### 4.9 Analysis of noise attack

**Fig 14:**
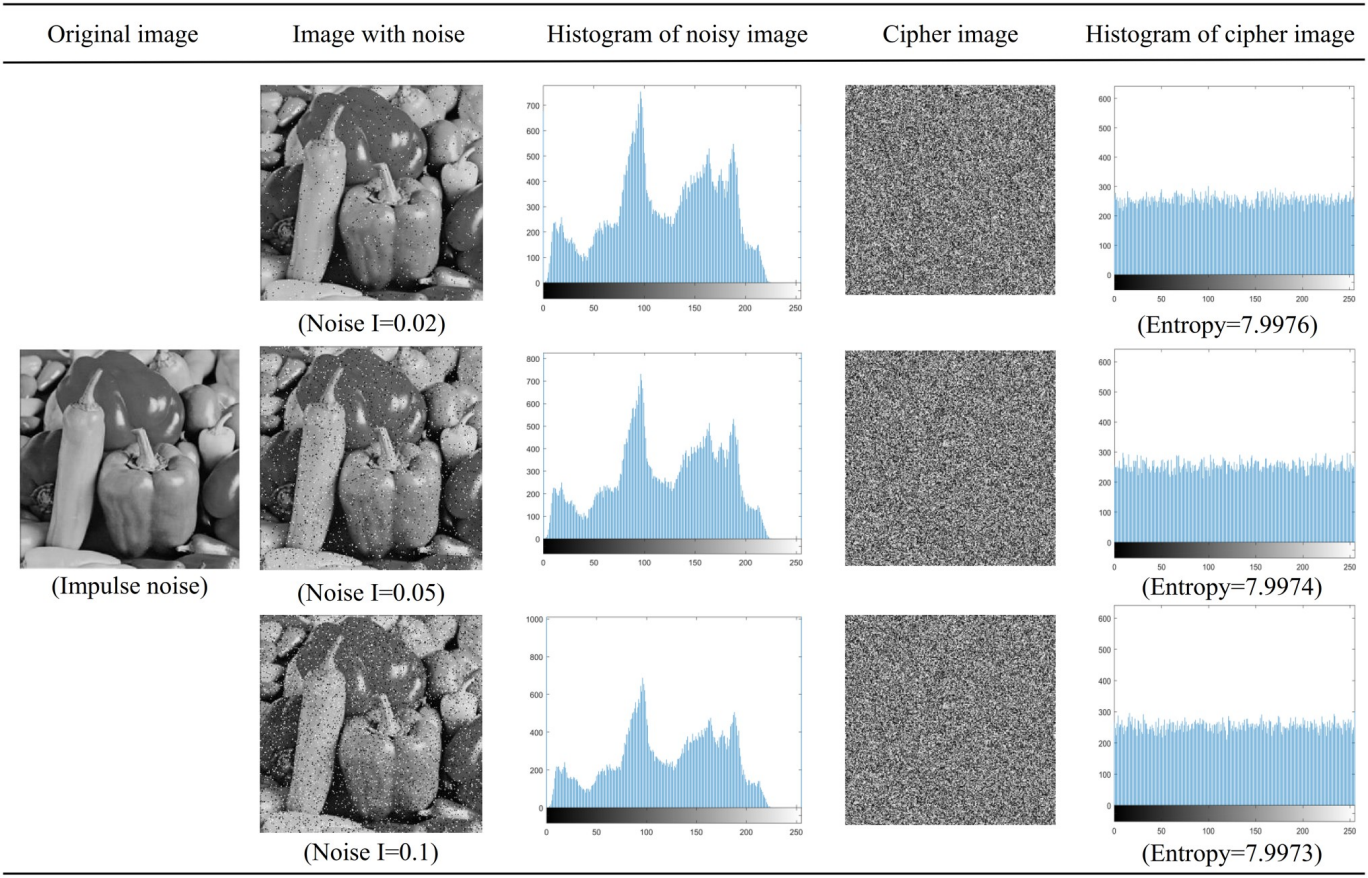
Histogram analysis and entropy analysis of salt-and-pepper noise image and encrypted image.

**Fig 15:**
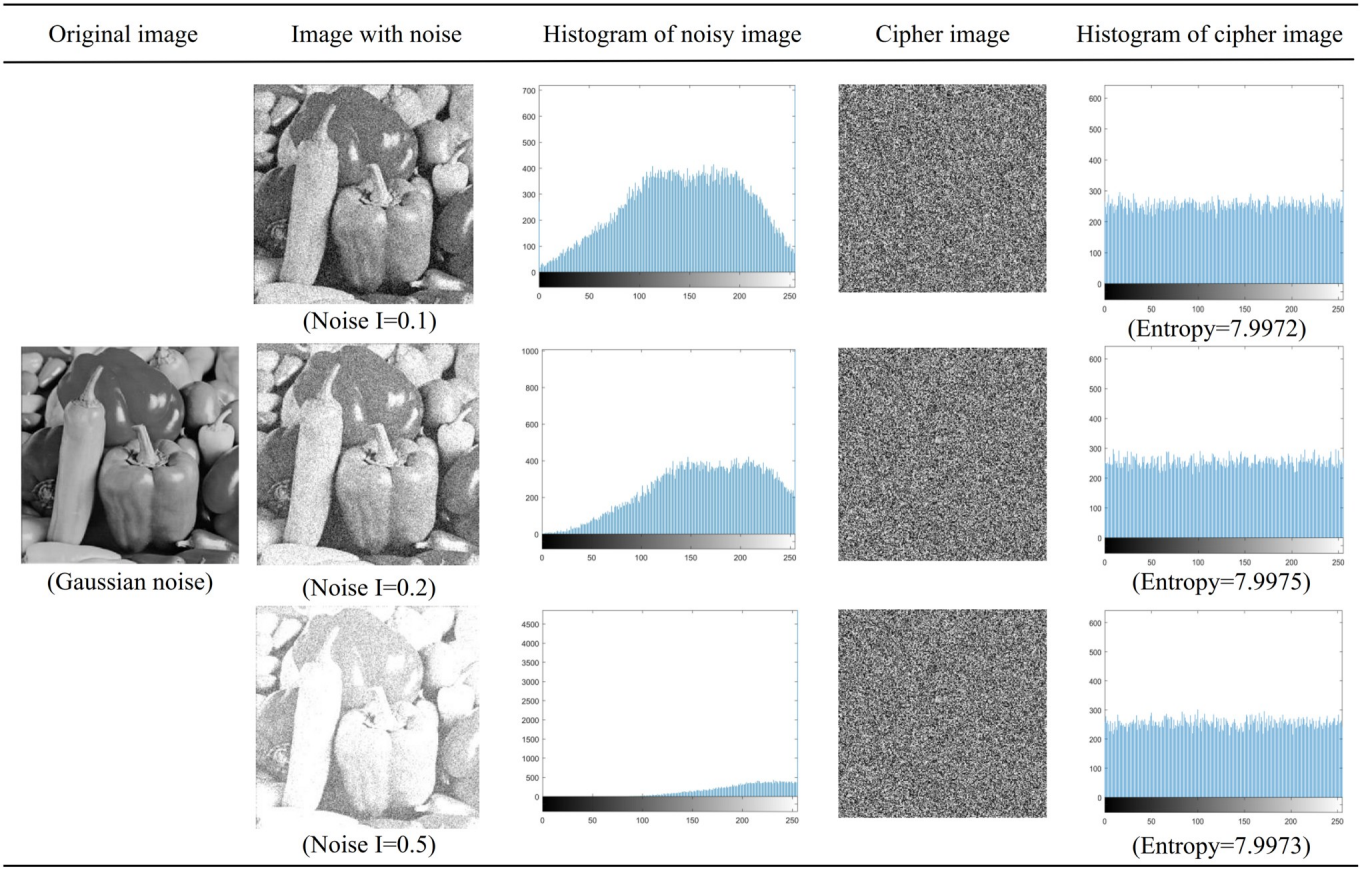
Histogram analysis and entropy analysis of Gaussian noise image and encrypted image.

When high-definition pictures and digital images are generated and information is transmitted to another terminal, various types of noise will interfere and affect the transmitted information, and the quality of the pictures will be severely distorted. This will have an adverse effect on subsequent image processing and image visual effects, and image noise will blur the image or even overwhelm the image features, bringing difficulties to the analysis. If a good cryptographic system is strong enough, it should be able to resist different types of noise to a certain extent. The algorithm in this chapter can encrypt images well even in the presence of noise. Table 10 shows the correlation coefficient analysis of Peppers image under the condition of noise. Figs 14 and 15 show that the distribution of salt-and-pepper noise is relatively sparse compared with Gaussian noise. Also, the correlation coefficient is very low, the histogram after encryption is evenly distributed, and the pixel value cannot be recognized by people.

**Table 10:** Correlation between adjacent pixels of noisy image.

Cipher Image		Horizontal direction	Vertical direction	Diagonal direction
Salt-and-pepper noise	I = 0.02	0.0029	-0.0133	0.0143
	I = 0.05	0.0031	-0.0035	-0.0046
	I = 0.1	0.0129	0.0190	0.0044
Gaussian noise	I = 0.1	-0.0023	0.0031	0.0436
	I = 0.2	0.0039	-0.0032	-0.0123
	I = 0.5	0.0062	-0.0102	0.0176

### 4.10 Algorithm and time complexity analysis

This article mainly introduces that can double scrambling-DNA closed-loop dynamic diffusion image encryption based on two-dimensional chaotic system (2D-LICM). Assuming that the size of the plaintext image is *M* × *N*, firstly, two sets of chaotic sequences are generated by the two-dimensional chaotic system (2D-LICM), the length is 4 × *M* × *N*, the algorithm complexity is *O*(4 × *M* × *N*). Secondly, the image is double-scrambling, the algorithm complexity is *O*(*M* × *N*), and then dynamic DNA coding and operation are carried out, including three DNA coding steps and two XOR operation steps, and its complexity is also *O*(4 × *M* × *N*). Based on the above analysis, the computational complexity of the algorithm is *O*(4 × *M* × *N*), which is a linear computational complexity, so the computational complexity of the algorithm depends on the size of the ordinary image.

The operation speed is an important characteristic parameter when the encryption algorithm meets the requirements of the security level. The experimental environment of this study includes MATLAB 2018b on Windows 10, Inter Core i5–6300HQ, 2.30GHz central processing unit (CPU) and 12.0GB random storage memory (RAM). Repeat the experiment 50 times, and then take the average of the above-mentioned experimental results as shown in the Table 11.

**Table 11:** Time consumption of encryption algorithms.

Algorithm	Computer configuration	Time(second)
Proposed	Core i5–6300HQ@2.3GHz CPU and 12GB RAM	5.1
Ref [1]	Core i7 3.4GHz and 8GB RAM	6.2
Ref [36]	Core i3–380M@2.53 GHz CPU and 4 GB RAM	6.580
Ref [37]	Core i7–3740QM@2.70 GHz CPU and 8GB RAM	5.3671

The image encryption scheme based on two-dimensional chaos in this paper is mainly composed of scrambling and diffusion processes. Therefore, the scrambling and diffusion and the generation of chaotic sequences directly affect the running time of the algorithm. Also, there are many factors that affect the time-consuming of encryption, including software and hardware environment, programming ability, programming language, and so on. It can be seen from Table 11 that compared with other similar algorithms, the encryption efficiency of this algorithm is the highest. In follow-up research, we can consider combining chaotic systems or DNA computing with other excellent encryption algorithms, which may have a qualitative leap in the field of image encryption in the future.

## 5. Discussion and prospects

Although some relatively good achievements have been achieved through the encryption scheme of this article, there are still shortcomings. With the gradual in-depth exploration of chaotic systems and DNA sequences, the research on image encryption based on chaotic systems and DNA coding still has a long way to go. This study has the following limitations:

1. Chaotic image encryption must consider the time complexity and space complexity of the algorithm, especially for the batch processing of data on a large-scale data platform, how to realize the fast processing of the algorithm will be very important.
2. This scheme only realizes encryption based on grayscale images.
3. This scheme only encrypts one image at a time.

In order to overcome these limitations, the focus of future work will be:

1. To strike a balance between the security of encryption and the complexity of the algorithm.
2. To extend the image encryption domain to other multimedia data such as color images, medical images, remote sensing images and other different types of images for further research to increase the applicability and practicability of the algorithm.
3. To consider the huge parallelism of DNA coding or use other encryption schemes to increase the encryption speed and realize the encryption of multiple images at the same time.

## 6. Conclusion

In this paper, we propose a dynamic update algorithm of double scrambling-DNA row and column closed loop based on image encryption. In the encryption process, the improved algorithm is proposed in both scrambling and diffusion stages. In the scrambling stage, the shortcoming of storing adjacent pixels in space when the Hilbert curve is used for scrambling is solved. The algorithm in this paper combines the Hilbert curve with Knuth-Durstenfeld shuffle algorithm to achieve double scrambling of the image and improve the data storage in memory effectiveness. In the diffusion stage, we improve the existing block closed-loop diffusion scheme and use the two-round diffusion of DNA encoding rows and columns. When the last line of ciphertext is generated, the first line of ciphertext is updated to realize the closed-loop dynamic update of the encryption system. The combination of DNA coding, plaintext, ciphertext and key stream increases the relationship among plaintext, ciphertext and key stream, and the inherent nonlinearity of DNA operation makes the input and output of encryption system not a simple linear relationship, which improves the sensitivity and security of encryption system. Because chaotic system is very sensitive to initial value, this paper designs a key stream generation algorithm using SHA-256. This algorithm greatly expands the key space, effectively resists violent attack, and still displays good encryption effect even when the original image contains noise. The simulation results and security analysis in the fourth section show that the encryption scheme proposed in this paper has strong reliability and security, so the algorithm has good application prospects.

## Supporting information

S1 File All the data for the experiments in the paper. (ZIP)
