# Supplementary material for: A double scrambling-DNA row and column closed loop image encryption algorithm based on chaotic system
Source: PLoS One. 2022 Jul 12;17(7):e0267094. doi: 10.1371/journal.pone.0267094 (PMC9275730; doi:10.1371/journal.pone.0267094)
Supplement: S1 File — (ZIP) [file pone.0267094.s001.zip › Supporting information/S1 File/Table.docx]

Table 1: DNA encoding rules.

| Rule | 1 | 2 | 3 | 4 | 5 | 6 | 7 | 8 |
| --- | --- | --- | --- | --- | --- | --- | --- | --- |
| A | 00 | 00 | 11 | 11 | 01 | 10 | 01 | 10 |
| G | 11 | 11 | 00 | 00 | 10 | 01 | 10 | 01 |
| C | 10 | 01 | 10 | 01 | 00 | 00 | 11 | 11 |
| T | 01 | 10 | 01 | 10 | 11 | 11 | 00 | 00 |

Table 2: DNA XOR operation.

| XOR | A | T | C | G |
| --- | --- | --- | --- | --- |
| A | A | T | C | G |
| T | T | A | G | C |
| C | C | G | A | T |
| G | G | C | T | A |

Table 3: Comparison of key spaces.

| Algorithm | Ref[27] | Ref[28] | Ref[29] | Ref[30] | Proposed |
| --- | --- | --- | --- | --- | --- |
| Key space | $2^{193}$ | $2^{189}$ | $2^{190}$ | $2^{256}$ | $2^{445}$ |

Table 4: Adjacent pixel correlation analysis of Peppers image.

| Direction | Proposed | Ref[30] | Ref[31] | Ref[32] |
| --- | --- | --- | --- | --- |
| Horizontal | **0.0039** | 0.0066 | 0.0068 | -0.0036 |
| Vertical | **0.0174** | 0.0261 | -0.0054 | 0.0023 |
| Diagonal | **-0.0034** | 0.0134 | 0.0010 | 0.0022 |

Table 5: Analysis and comparison of global entropy and local entropy between original image and encrypted image.

| Test Image | Global entropy | | | | Local entropy ($k$,$T_{B}$,$\alpha$)=(30,1936,0.001) | | |
| --- | --- | --- | --- | --- | --- | --- | --- |
|  | Plain image | Ref[33] | Proposed |  | Plain image | Ref[33] | Proposed |
| Baboon | 7.3715 | 7.9971 | 7.9973 |  | 6.7807 | **7.9021** | **7.9028** |
| Barbara | 6.5838 | 7.9973 | 7.9984 |  | 6.7223 | 7.9014 | **7.9027** |
| Boat | 7.1612 | 7.9971 | 7.9993 |  | 6.3282 | **7.9029** | 7.9035 |
| Couple | 6.1689 | 7.9970 | 7.9970 |  | 6.3158 | 7.8987 | **7.9020** |
| Chemical plant | 7.0193 | 7.9973 | 7.9982 |  | 6.6642 | 7.9002 | **7.9025** |
| Clock | 7.2943 | 7.9954 | 7.9989 |  | 6.4612 | **7.9023** | **7.9022** |
| Elaine | 7.4874 | 7.9971 | 7.9980 |  | 6.4139 | **7.9020** | **7.9025** |
| Fingerprint | 6.5945 | 7.9971 | 7.9979 |  | 7.2007 | **7.9029** | **7.9024** |
| Gold Hill | 7.4460 | 7.9975 | 7.9981 |  | 6.4601 | **7.9020** | **7.9026** |
| Peppers | 7.3797 | 7.9970 | 7.9974 |  | 6.4479 | 7.9043 | **7.9024** |
| Plane | 6.3908 | 7.9973 | 7.9991 |  | 6.1305 | **7.9016** | 7.9037 |
| Resolution chat | 7.4590 | 7.9963 | 7.9971 |  | 7.0599 | 7.9035 | **7.9028** |
| MEAN | 7.0297 | 7.9970 | 7.9981 |  | 6.5821 | **7.9020** | **7.9027** |
| PASS/ALL | - | - | - |  | - | 7/12 | 10/12 |

Table 6: Black and white correlation coefficient and entropy.

| Image | Entropies | Correlation coefficients | | |
| --- | --- | --- | --- | --- |
|  |  | Horizontal | Vertical | Diagonal |
| Full black | 0 | - | - | - |
| Cipher image of black | 7.9970 | 0.0211 | -0.0125 | -0.0134 |
| Full white | 0 | - | - | - |
| Cipher image of white | 7.9972 | -0.0306 | 0.0057 | -0.0247 |

Table 7: Comparison of NPCR and UACI.

| Image | NPCR | | | | UACI | | | |
| --- | --- | --- | --- | --- | --- | --- | --- | --- |
|  | Proposed | [25] | [26] | [30] | Proposed | [25] | [26] | [30] |
| Baboon | 99.6353 | 96.8450 | 99.6143 | 99.6348 | 33.4898 | 32.4069 | 33.4675 | 33.4725 |
| Peppers | 99.6387 | 99.0281 | 99.6135 | 99.6348 | 33.5359 | 33.1006 | 33.4692 | 33.3637 |
| Plane | 99.6248 | 98.5591 | 99.6226 | 99.5855 | 33.5068 | 33.1368 | 33.4251 | 33.5052 |

Table 8 : Comparison of MAE, MSE and PSNR.

| Image | Plain-encrypted images | | | After one bit changed in plain images | | |
| --- | --- | --- | --- | --- | --- | --- |
|  | MAE | MSE | PSNR | MAE | MSE | PSNR |
| Peppers | 73.6438 | 7849.3 | 9.3681 | 73.4192 | 7815.1 | 9.3422 |
| Baboon | 71.3447 | 7343.9 | 9.4715 | 71.2780 | 7319.4 | 9.4860 |
| Terrace | 87.3575 | 11445.6 | 7.5444 | 87.2861 | 77414.9 | 7.5561 |
| Plane | 83.8329 | 10089.3 | 7.3979 | 83.6259 | 10076.2 | 7.3983 |
| Cameramen | 79.5888 | 9422.0 | 8.3894 | 79.5472 | 9438.5 | 8.3818 |
| Couple | 84.2192 | 9462.3 | 7.8686 | 84.2471 | 9463.9 | 7.8616 |

Table 9: Difference rates between two images encrypted by slightly different keys.

| Secret keys | Difference rates (%) | | | | | |
| --- | --- | --- | --- | --- | --- | --- |
|  | Peppers | Baboon | Terrace | Plane | Cameramen | Couple |
| $key_{1}$( $x_{0}^{'}=x_{0}+10^{-13}$) | 99.60 | 99.61 | 99.62 | 99.58 | 99.58 | 99.63 |
| $key_{2}$( $y_{0}^{'}=y_{0}+10^{-13}$) | 99.59 | 99.62 | 99.58 | 99.61 | 99.57 | 99.61 |
| $key_{3}$( $S1^{'}=S1+10^{-13}$) | 99.61 | 99.61 | 99.59 | 99.56 | 99.60 | 99.62 |
| $key_{4}$( $S2^{'}=S2+10^{-13}$) | 99.63 | 99.59 | 99.60 | 99.60 | 99.61 | 99.63 |
| $key_{5}$( $S3^{'}=S3+10^{-13}$) | 99.63 | 99.60 | 99.59 | 99.61 | 99.61 | 99.62 |
| $key_{6}$( $S4^{'}=S4+10^{-13}$) | 99.62 | 99.61 | 99.61 | 99.58 | 99.60 | 99.59 |

Table 10: Correlation between adjacent pixels of noisy image.

| Cipher image | | Horizontal direction | Vertical direction | Diagonal direction |
| --- | --- | --- | --- | --- |
| Salt-and-pepper noise | I=0.02 | 0.0029 | -0.0133 | 0.0143 |
|  | I=0.05 | 0.0031 | -0.0035 | -0.0046 |
|  | I=0.1 | 0.0129 | 0.0190 | 0.0044 |
| Gaussian noise | I=0.1 | -0.0023 | 0.0031 | 0.0436 |
|  | I=0.2 | 0.0039 | -0.0032 | -0.0123 |
|  | I=0.5 | 0.0062 | -0.0102 | 0.0176 |

Table 11: Time consumption of encryption algorithms.

| Algorithm | Computer configuration | Time(second) |
| --- | --- | --- |
| Proposed | Core i5-6300HQ@2.3GHz CPU and 12GB RAM | 5.1 |
| [1] | Core i7 3.4GHz and 8GB RAM | 6.2 |
| [36] | Core [i3-380M@2.53 GHz](mailto:i3-380M@2.53%20GHz) CPU and 4 GB RAM | 6.580 |
| [37] | Core i7-3740QM@2.70 GHz CPU and 8GB RAM | 5.3671 |
